# Polymorphic SERPINA3 prolongs oligomeric state of amyloid beta

**DOI:** 10.1371/journal.pone.0248027

**Published:** 2021-03-04

**Authors:** Maruf Mohammad Akbor, Nobuyuki Kurosawa, Hiroki Nakayama, Ayumi Nakatani, Koji Tomobe, Yoichi Chiba, Masaki Ueno, Masashi Tanaka, Yasuyuki Nomura, Masaharu Isobe

**Affiliations:** 1 Department of Life Sciences and Bioengineering, Laboratory of Molecular and Cellular Biology, Faculty of Engineering, University of Toyama, Toyama, Japan; 2 Department of Pathophysiology, Yokohama University of Pharmacy, Yokohama, Japan; 3 Department of Pathology and Host Defense, Faculty of Medicine, Kagawa University, Miki-cho, Kita-gun, Kagawa, Japan; 4 Department for Health and Longevity Research, National Institutes of Biomedical Innovation, Health and Nutrition, Shinjuku, Tokyo, Japan; 5 Department of Pharmacology, School of Medicine, Kurume University, Kurume, Fukuoka, Japan; National Center for Geriatrics and Gerontology, JAPAN

## Abstract

Molecular chaperon SERPINA3 colocalizes with accumulated amyloid peptide in Alzheimer’s disease (AD) patient’s brain. From the QTL analysis, we narrowed down Serpina3 with two SNPs in senescence-accelerated mouse prone (SAMP) 8 strain. Our study showed SAMP8 type Serpina3 prolonged retention of oligomeric Aβ 42 for longer duration (72 hr) while observing under transmission electron microscope (TEM). From Western blot results, we confirmed presence of Aβ 42 oligomeric forms (trimers, tetramers) were maintained for longer duration only in the presences of SAMP8 type Serpina3. Using SH-SY5Y neuroblastoma cell line, we observed until 36 hr preincubated Aβ 42 with SAMP8 type Serpina3 caused neuronal cell death compared to 12 hr preincubated Aβ 42 with SAMR1 or JF1 type Serpina3 proteins. Similar results were found by extending this study to analyze the effect of polymorphism of *SERPINA3* gene of the Japanese SNP database for geriatric research (JG-SNP). We observed that polymorphic SERPINA3 I308T (rs142398813) prolonged toxic oligomeric Aβ 42 forms till 48 hr in comparison to the presence wild type SERPINA3 protein, resulting neuronal cell death. From this study, we first clarified pathogenic regulatory role of polymorphic SERPINA3 in neurodegeneration.

## Introduction

Alzheimer’s disease (AD) leads to the progressive neurodegeneration especially in brain regions important for cognitive function. Over the last two decades researchers have been pushing ahead to decipher underlying patho-physiological mechanisms of late onset Alzheimer disease (LOAD) but still in maze due to involvement of multifactorial genes [1–3]. Even until now aging is the most substantial risk factor for LOAD [4, 5].

Senescence-accelerated mouse prone 8 (SAMP8) strain become an acceptable rodent model for the earliest cognitive changes associated with Alzheimer’s disease [6–9]. SAMP8 strain exhibits severe age-related learning and memory deficits (LMD) at 2 months of age, which further aggravates with advancing age without displaying any other signs of premature aging [10–12]. Although some studies provide insight into the mechanisms that contribute to learning and memory decline in SAMP8 mice, but no causative genes were evidently identified yet [9, 13]. With the advantages of quantitative trait linkage (QTL) analysis, our group identified five loci involved in LMD phenotype by step-through passive avoidance test with 264 F2 intercross SAMP8 x Japanese fancy mouse (JF) 1 mice [14]. JF1 strain originated from the Japanese wild mouse, *Mus musculus molossinus* showing normal learning and memory function, used as genetically distinct control strain [15]. Senescence-accelerated-resistant (SAMR) 1 mouse considered as an internal control strain of SAMP8, because of their common genetic background [16]. Polygenic inheritance of SAMP8 mice underlie involvement of multiple genes in the development of its LMD [17]. Previously we proposed, *Hcn1* gene as a candidate on chromosome 13 LMD locus for possible involvement of learning and memory dysfunction in SAMP8 strain [18]. Besides, chromosome 12 LMD locus contributes most strongly on short retention time in step-through passive avoidance test, therefore exacting further extensive study. By combining QTL and transcriptomic approaches, here we first provided evidences of Serine protease inhibitor (Serpin) a3n from SAMP8 strain as a pathogenic chaperon of amyloid peptide pathogenesis.

The predominant toxic peptide, amyloid beta (Aβ) 42 is the proteolytic fragment of amyloid precursor protein (APP) [19]. Aβ 42 undergoes complex multistep conformational changes from monomer, oligomer, protofibrils, and fibrils finally ending up as accumulated plaque form [20, 21]. Various reports suggested that soluble oligomeric Aβ 42 are substantially more toxic to neurons or neuronal like cells leading to cell death compared to insoluble protofibrils & fibrils but no direct evidences were established to functionally correlate such events with AD [22–24]. Neurotoxic oligomers are composed to loosely aggregated strands without characteristic cross-beta sheet structure of fibrils, proposed as a potential contributor of the initiation of AD pathogenesis [20, 25–28].

In the present study, we showed SAMP8 type Serpina3n enhanced the toxic Aβ 42 peptide states especially retention of transient oligomers for delayed time (72 hr) while observing under transmission electron microscope (TEM). We then extended our study to analyze polymorphism of *SERPINA3* gene found in the Japanese SNP database for geriatric research (JG-SNP) [29]. We found that polymorphic SERPINA3 I308T (rs142398813) could also prolong toxic oligomeric Aβ 42 triggering neuronal cell death using neuroblastoma SH-SY5Y cell line, thus act as pathogenic chaperon protein. This is the first study which successfully established direct correlation among pathogenic chaperon SERPINA3, Aβ 42 toxic form associated with neuronal cell death and AD pathogenesis.

We also observed spongy like degenerative neuronal structures in SAMP8 mouse brain stem with colocalized Serpina3 protein expression, might leads to its early cognitive deficits indicated pathogenic role of this chaperon protein [11, 12, 17]. There are several rodent models of early onset of Alzheimer disease (EOAD) or Familial Alzheimer disease (FAD) but no acceptable model mouse was yet proposed for LOAD. Hence, our study results supported to propose SAMP8 mice as a promising rodent model for studying Aβ oligomer induced neurodegenerative disease like age related AD type dementia especially LOAD [6, 7, 13, 30, 31].

## Results

### QTL analysis of chromosome 12 identified SAMP8 type *Serpina3* as a candidate

**Fig 1:**
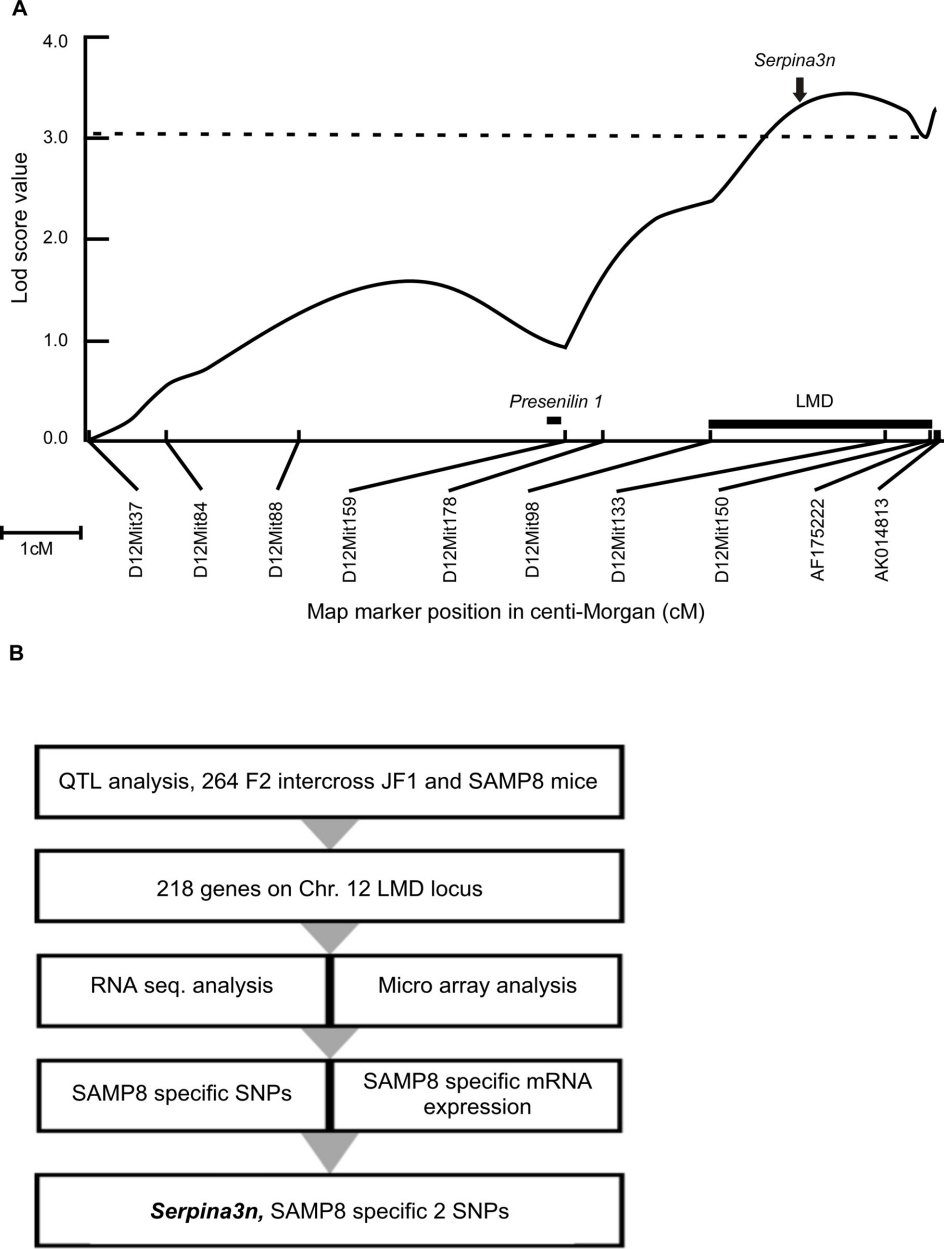
QTL analysis to identify candidate gene on chromosome 12 LMD locus. (A) Lod (logarithm of odd) score plots for LMD phenotype on chromosomes 12. *X*-axis: Indicates map position in centi-Morgan (cM). Microsatellite markers are indicated. *Y-*axis: Lod scores for QTL in (SAMP8 x JF1) F2 progeny. Vertical dotted line on lod plot indicates the lod = 3.1 significance threshold. Both male and female scores were standardized and analyzed by Mapmaker/QTL. Thick small horizontal line indicates the 1-lod support interval for the noted LMD locus. Solid arrow indicates position of *Serpina3n* (104,406,729–104,414,329 bp), vertical bar indicates position of *Presenilin 1* (83,688,152–83,735,199 bp). (B) Strategy to narrow down SAMP8 specific SNPs.

To narrow down chromosome 12 LMD region and assess candidate genes, linkage analysis with additional markers were performed placing LMD locus (15.40 Mb) in between D12Mit98 (100.5 Mb) and D12Mit150 (115.9 Mb) markers (Fig 1A). By combining, RNA-seq and micro-array analysis on this LMD locus, *Alpha1 anti-chymotrypsin (ACT) or Serpina3n* gene was narrowed down out of 218 genes (Fig 1B). *Serpina3n* was co-positioned to the apogee of logarithm of odd ratio (lod) score (3.54) in LMD plot (Fig 1A). We first compared expression level of genes located in this LMD locus. Among the 218 genes, we found elevated expression of Serpina3n in SAMP8 mice compared to that of JF1 and SAMR1 types while expression of homologous as well other genes in the LMD locus were not affected among strains. By using microarray data, comparison of mRNA expression profile of Serpina family genes clustered in LMD region was shown in S1A Fig. Besides, gene expression from RNA-seq data showed increased level of SAMP8 type *Serpina3n* in this LMD region (S1B Fig). We also found one synonymous and two non-synonymous polymorphisms specific to SAMP8 type Serpina3n (S1C Fig). These two SAMP8 specific polymorphisms (M273L, K281R) differed from SAMR1 and JF1 types Serpina3n whereas additional six polymorphisms were common both in SAMP8 and SAMR1 compared to JF1 type Serpina3n (S1 Table).

### SAMP8 Serpina3 SNP deters formation of Aβ 42 fibers

To analyze the functional differences of this polymorphic Serpina3n, we expressed recombinant wild type and polymorphic proteins using mammalian cell line Expi293F. We observed higher size bands (around 50–65 kDa) compared to expected size (47 kDa) of expressed recombinant mouse Serpina3 proteins (S1E Fig, lanes 2–4).

**Fig 2:**
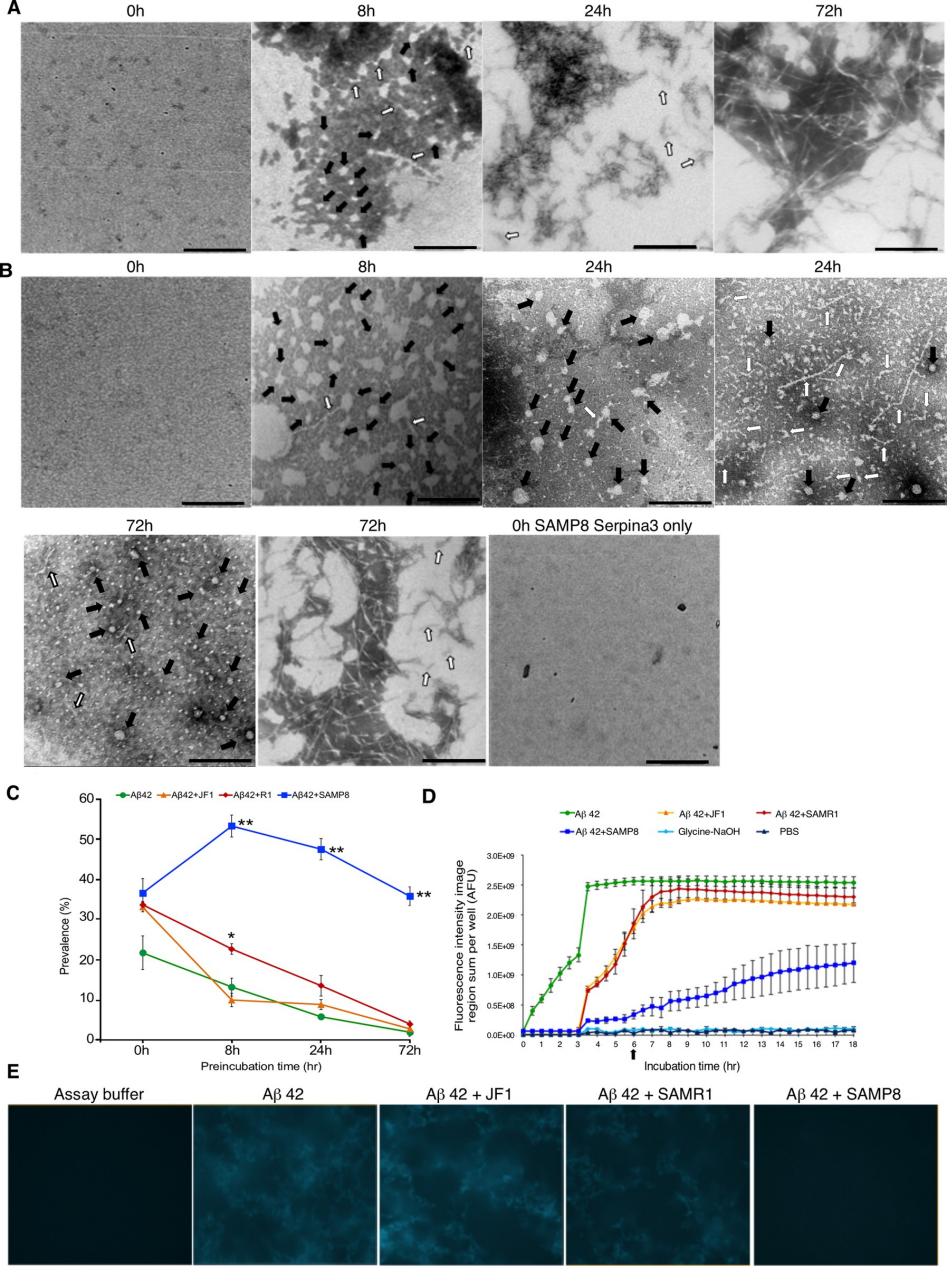
Regulation of various Aβ peptide forms by mouse polymorphic Serpina3n. Representative TEM images of Aβ peptide at different preincubation time points using (A) Aβ 42 peptide. (B) Aβ 42 + SAMP8 Serpina3n. 0 hr preincubation did not show any define structure either alone or in presence of both Aβ peptide with SAMP8 Serpina3n protein. Solid arrow indicated oligomeric peptide, while open arrow indicated protofibrillar form of Aβ peptide. Representative fibrillar form of Aβ peptides were shown at 24 hr (Fig 2A) and 72 hr (Fig 2A and 2B) of preincubation. Scale bar: 100 nm. (C) Kinetics of amyloid β peptide oligomerization under influence of polymorphic mouse Serpina3n. Here, x-axis shows prevalence of oligomer rich observation fields (in percentage) and y-axis shows preincubation time (hr). Significance levels were tested SAMP8 vs. SAMR1; SAMP8 vs. JF1; SAMR1 vs. JF1. (n = 3, * *P* ≤ 0.05; ** *P* ≤ 0.01. One-way ANOVA with Tukey and Kramer’s honestly significance difference test was used). (D) Thioflavin T fluorescence assay (data represent Mean ± SEM, n = 3). AFU refers arbitrary fluorescence units. (E) Representative images of thioflavin T fluorescence assay at 6 hrs.

**Fig 3:**
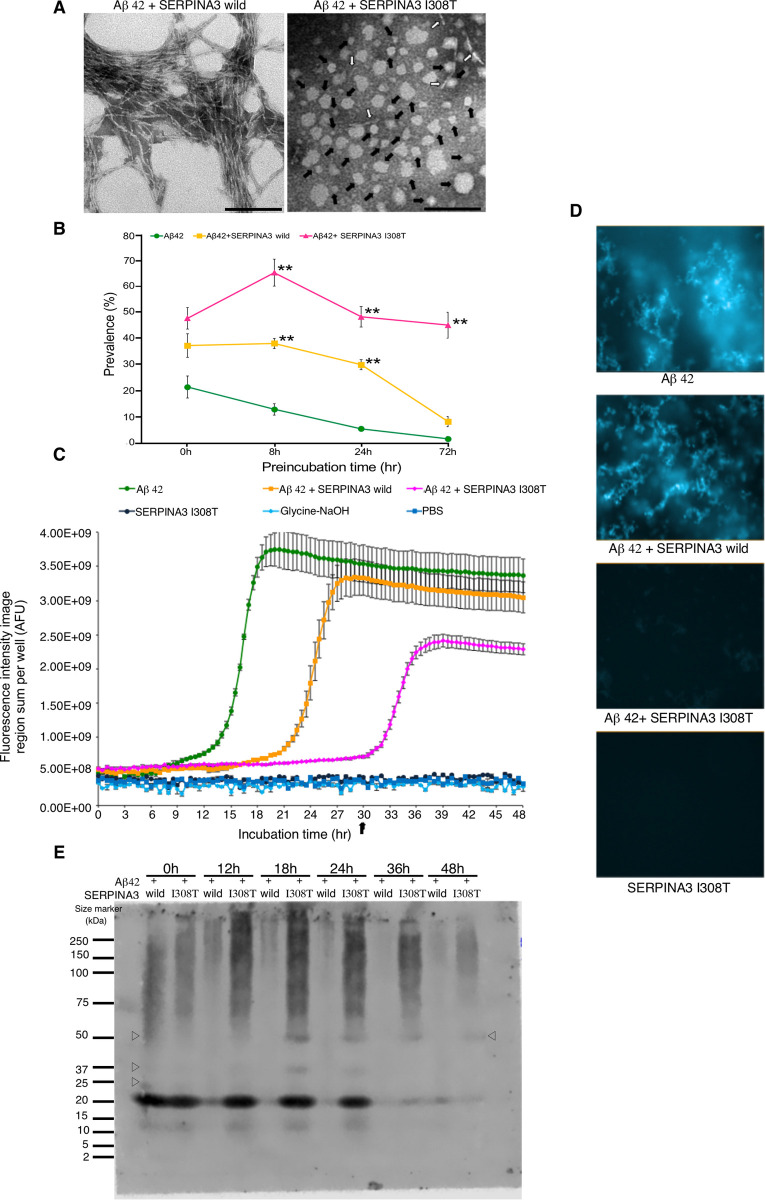
Regulation of various Aβ peptide forms by human polymorphic SERPINA3. (A) Representative TEM images of Aβ peptide at 72 hr preincubation. Typical fibrillar form was observed in Aβ 42 + SERPINA3 wild type; whereas Aβ 42 + SERPINA3 I308T still showed irregularly shaped oligomers as indicated by solid arrow and while open arrow indicated protofibrillar form. Scale bar: 100 nm. (B) Kinetics of amyloid β peptide oligomerization in presence of human SERPINA3. Here, x-axis shows prevalence of oligomer rich observation fields (in percentage) and y-axis shows preincubation time (hr). Significance levels were tested Aβ 42 + SERPINA3 I308T vs. Aβ 42 + SERPINA3 wild; Aβ 42 + SERPINA3 I308T vs. Aβ 42; Aβ 42 + SERPINA3 wild vs. Aβ 42. (n = 3, ** *P* ≤ 0.01. One-way ANOVA with Tukey and Kramer’s honestly significance difference test was used). (C) Thioflavin T fluorescence assay (data represent Mean ± SEM, n = 3). AFU refers arbitrary fluorescence units. (D) Representative images of thioflavin T fluorescence assay at 30 hrs. (E) Representative image of gradient gel native PAGE, showing Aβ 42 peptide in presence of human SERPINA3 recombinant proteins, at 0 to 48 hrs of preincubation. Open triangle showed presence of oligomeric Aβ peptide conformations. See also in S3 Fig.

Using transmission electron microscope, we deduced amorphous, irregularly shaped oligomers (considering round, ellipsoidal, circular with protruding small branches etc.), protofibrils and fibrillar forms of amyloid peptide in different preincubation time courses in the presence or absence of SERPINA3 proteins (Figs 2 and 3). Preincubated sample at 0 hr showed no define amyloid peptide conformation considered as amorphous form. Representative images of irregularly shaped oligomers marked with solid arrows and small protofibrils protruding from oligomers or large protofibrils marked with open arrows. Mature fibrils were aggregated as final stable form of amyloid beta peptide shown in Figs 2A and 2B and 3A. 0 hr preincubated samples of SAMP8 Serpina3 protein showed no define structures same like Aβ 42 peptide as shown in Fig 2B. We observed that dark staining area containing oligomers tend to decrease with longer preincubation time courses. Prevalence frequencies were calculated by tallying various physical states of Aβ 42 peptide considering single observation field as 500 nm scale (see Materials and Methods). Representative TEM images of preincubated Aβ 42 only and SAMP8 Serpina3 with Aβ 42 were shown respectively in Fig 2A and 2B. At 24 hr preincubation, Aβ 42 alone can be observed as protofibrils and fibrils, whereas Aβ 42 with SAMP8 type Serpina3 showed presence of oligomeric rich field as well as mixture of protofibril with oligomeric Aβ peptide. Besides, by 72 hr preincubation, SAMP8 type Serpina3 still showed presence of oligomers in addition of fibrils and protofibrillar mixture of Aβ 42 peptide. Contrarily Aβ 42 alone converted into mature fibrils by this time course. Kinetics of oligomerization was presented in Fig 2C. From these results, we found that active SAMP8 type Serpina3 showed highest prevalence of oligomeric amyloid beta at 8 hr of preincubation and maintained toxic oligomeric form till 72 hr whereas major portion of Aβ converted to mature fibrils by 24 hr in the case of Aβ 42 alone or in combination with SAMP8 inactive form, SAMR1, or JF1 type Serpina3 proteins.

To support these findings, we investigated the effects of recombinant Serpina3 proteins on the fibrillization process of Aβ 42 through thioflavin T (ThT) fluorescent assay. With the advantage of high content image analysis system Operetta, we monitored fluorescence levels of thioflavin T dye incorporating into large protofibrillar and fibrillar forms of Aβ peptide. We observed that fibrillization process was faster in case of Aβ 42 peptide only, reached to fluorescence plateau quickly by 3 to 4 hrs, followed by JF1 and SAMR1 type Serpina3 proteins of 5 to 7 hrs, whereas SAMP8 derived Serpina3 protein could maintain oligomeric or small protofibrils longer duration like 13 hr. The signal at plateau obtained in case of Aβ 42 with SAMP8 Serpina3 was nearly half of the rest of samples (Aβ 42 alone, Aβ 42 with JF1 or SAMR1 Serpina3 proteins) (Fig 2D). Representative images captured during ThT assay period of 6 hr were shown in Fig 2E. We found Aβ 42 alone, Aβ 42 with JF1 or SAMR1 Serpina3 proteins mostly appeared as fibrillar Aβ 42 by this time point whereas Aβ 42 with SAMP8 Serpina3 proteins still showing absence of any kind aggregated Aβ peptide.

Control sample (Glycine NaOH, pH8.5; PBS pH7.4) showed no thioflavin T fluorescence signals as background. Aβ 42 with inactive form of SAMP8 Serpina3 resulted earlier fibrillization process than that of active form and behaved same as like only Aβ 42 sample (S2A Fig). To ask whether the polymorphism of Serpina3n behaves dominantly or recessively, we performed thioflavin T assay using equimolar mixture of SAMP8 and JF1 types proteins. We observed that JF1 type Serpina3n accelerated Aβ 42 peptide fibrillization process whereas SAMP8 type alone as well as mixture of SAMP8 and JF1 types Serpina3n delayed fibrillization in similar pattern suggesting dominant effect of the polymorphism (S2B Fig).

To confirm presence of low molecular weight (LMW) oligomeric Aβ 42 in absence or presence of serpina3 proteins, we performed Western blot assays using 6% to 20% gradient gels. To check presence of oligomeric seed in our Aβ 42 preparation, gradient type sodium dodecyl sulfate polyacrylamide gel electrophoresis (SDS-PAGE) was used to obtain accurate molecular weight (see Materials and Methods). We compared the results of SDS-PAGE with native PAGE at 0 hr preincubation time point and found that band near 20 kDa size marker in native PAGE actually comprises three different bands like monomer (4.5 kDa), trimer (13.5 kDa) and tetramer (18 kDa) of SDS-PAGE as shown in S3C Fig. Since SDS-PAGE was not suitable to analyze the conformational changes of Aβ peptide, we performed further analysis using native PAGE. Then, we found SAMP8 type serpina3 prolonged presence of Aβ 42 peptide band near 20 kDa till 48 hr as shown in S3A and S3B Fig. During preincubation periods, Aβ 42 band near 20 kDa mostly disappeared at 18 hr in the absence or presence of serpina3 from JF1 or SAMR1 types due to formation of high molecular weight Aβ 42 fibrils. These results clearly supported that only active form of polymorphic SAMP8 Serpina3 could maintain toxic oligomeric form of Aβ 42 for delayed time periods compared to Aβ 42 alone or in mixture with JF1 or SAMR1 or inactive form of SAMP8 type Serpina3 proteins.

To find out the cause of SAMP8 Serpina3 driven delayed Aβ peptide fibrillization, we examined the interactions between mouse Serpina3n with Aβ 42 peptide using pull-down assay. Our results indicated that at 72 hr, only SAMP8 Serpina3 was coprecipitated with biotinylated Aβ 42 whereas JF1 and SAMR1 types Serpina3 did not, while Serpina3 of each strain was present in binding with Aβ 42 till 24 and 48 hrs (S3D Fig).

Similar way, we compared the kinetics of Aβ 42 peptide oligomerization in presence of wild type and polymorphic human SERPINA3 proteins under TEM. TEM observation showed that polymorphism of human SERPINA3 at Isoleucine 308 Threonine could maintain oligomeric form of amyloid peptide compared to wild type human SERPINA3 or Aβ 42 alone till 72 hr (Fig 3A and 3B). Representative images of this TEM observation for 72 hr were shown in Fig 3A. From the kinetic graph, it was observed that polymorphic SERPINA3 I308T (rs142398813) always maintained 45 ~ 55% of oligomeric Aβ compared to that of wild type (Fig 3B). From additional supports of ThT fluorescence assay, we observed human SERPINA3 I308T (rs142398813) could maintain oligomeric form of Aβ compared to Aβ 42 with SERPINA3 wild type. Besides, polymorphic SERPINA3 I308T (rs142398813) regulated Aβ fibrillization did not reach to highest plateau level like Aβ 42 only or when incubated with wild type SERPINA3, indicating a window of oligomeric Aβ retention for longer duration. In absence of Aβ 42 peptide, SERPINA3 I308T (rs142398813) did not show any kind of fluorescence signal behaved like control assay buffers as shown in Fig 3C. Representative images captured during ThT assay period of 30 hr showed that Aβ 42 alone, Aβ 42 with SERPINA3 wild were mostly converted to fibrils whereas SERPINA3 I308T (rs142398813) could keep oligomeric or small protofibrillar Aβ peptide as shown in Fig 3D.

Similar way using gradient gel native PAGE, we confirmed human SERPINA3 I308T prolonged presence of Aβ 42 band near 20 kDa and was strongly observed till 24 hr which continued till 48 hr faintly. Two additional bands were detected around 50 kDa (from 18 to 48 hrs) and 37 kDa (18 to 24 hrs). In the presence of SERPINA3 wild type Aβ 42 band near 20 kDa mostly disappeared at 18 hr due to the formation of high molecular weight Aβ 42 fibrils as shown in Fig 3E.

### Polymorphic SERPINA3 prolongs toxic oligomeric Aβ 42 causing neuronal cell death

**Fig 4:**
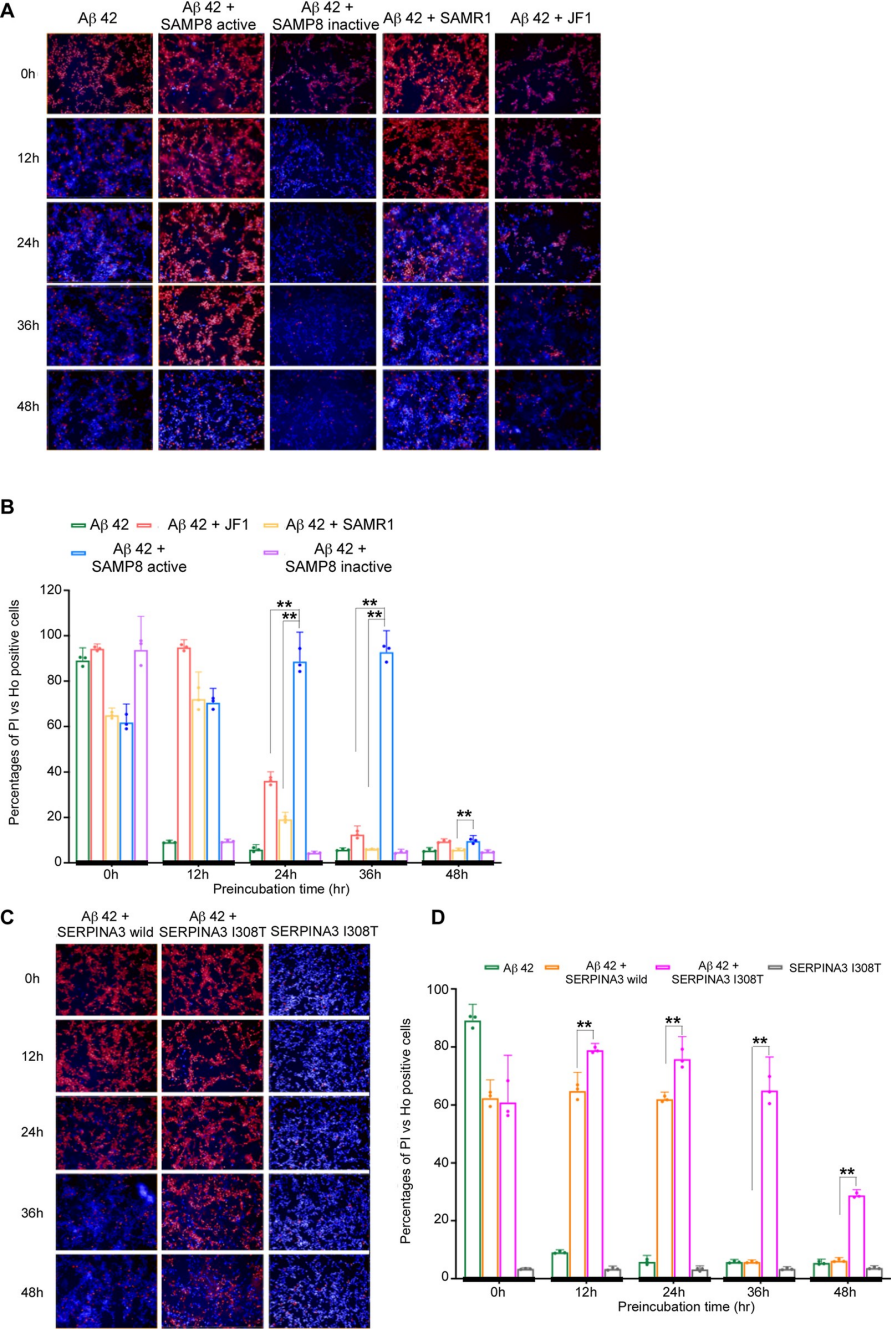
Aβ 42 induced cytotoxicity using SHSY5Y neuroblastoma cell line. Hoechst 33342 showing blue signal of intact nucleus; propidium iodide (PI) identified red signal of damaged cell DNA. (A) Representative images of cytotoxicity assay at different preincubation time points using mouse Serpina3n. (B) Bar graphs showing percentage ratio of PI vs. Hoechst 33342 positive cells in y-axis at different preincubated time points indicated with thick vertical line in x-axis. Significance levels were tested SAMP8 vs. SAMR1 & SAMP8 vs. JF1. (n = 3, * *P* ≤ 0.05; ** *P* ≤ 0.01. One-way ANOVA with Tukey and Kramer’s honestly significance difference test was used). (C) Representative images at different preincubation time points using human SERPINA3. (D) Bar graphs showing percentage ratio of PI vs. Hoechst 33342 positive cells. Significance levels were tested against human SERPINA3 I308T vs. SERPINA3 wild type. (n = 3, * *P* ≤ 0.05; ** *P* ≤ 0.01. Tukey and Kramer’s honestly significance difference test was used).

In the course of examining oligomeric Aβ induced cytotoxicity, we observed that active SAMP8 Serpina3 with Aβ 42 could cause neuronal SH-SY5Y cell death through exposure of toxic oligomeric form until 36 hr of preincubation compared to that of Aβ 42 alone or with SAMP8 inactive, SAMR1 or JF1 type Serpina3 proteins. Representative images of different preincubated samples causing neuronal cell death were shown in Figs 4A and 4B and S4A, S4C, S4E and S4F. We stained dead cells with Propidium Iodide (PI) (red signal) and live cells with Hoechst 33342 (blue signal) and calculated the percentage ratio of PI vs Hoechst 33342 positive cells, then plotted against different preincubation time courses as shown in Fig 4B. The results also supported that Aβ 42 with active SAMP8 Serpina3 could cause cell death till 36 hr of preincubation. Simultaneous experimental results of LDH assay further concur our findings as shown in S4A Fig.

While executing human SERPINA3 regulated oligomeric Aβ induced cytotoxicity, polymorphic SERPINA3 I308T (rs142398813) with Aβ 42 caused neuronal cell death till 48 hr of preincubation whereas SERPINA3 wild type showed such till 24 hr (Fig 4C and 4D). Assertive results of LDH assay were presented in S4B Fig. Preincubation of human SERPINA3 I308T (rs142398813) without Aβ 42 peptide showed no cytotoxic effects towards neuronal SH-SY5Ycell line imitates control samples (culture medium or PBS with culture medium) as shown in Figs 4C and 4D and S4B, S4D–S4F. All the samples were triplicated and results were found statistically significant. Axonal integrity was again checked using anti tubulin beta III to stain microtubules of axons. We observed, in case of SAMP8 type active Serpina3 proteins until 36 hr of preincubated Aβ 42 dismantled axons as shown by dispersed green signals. Whereas 48 hr preincubated Aβ 42 with polymorphic human SERPINA3 I308T (rs142398813) was still showing degeneration of axons. Representative images were shown in S4C, S4D and S4F Fig. These results also supported our earlier observations that SAMP8 Serpina3 could maintain oligomeric Aβ till 36 hr of preincubation where as human SERPINA3 I308T (rs142398813) maintained till 48 hr of preincubation resulting damaged axonal structures compared to controls (Figs 4A–4D and S4A–S4F).

### Serpina3 colocalizes with degenerated brain regions in mouse

**Fig 5:**
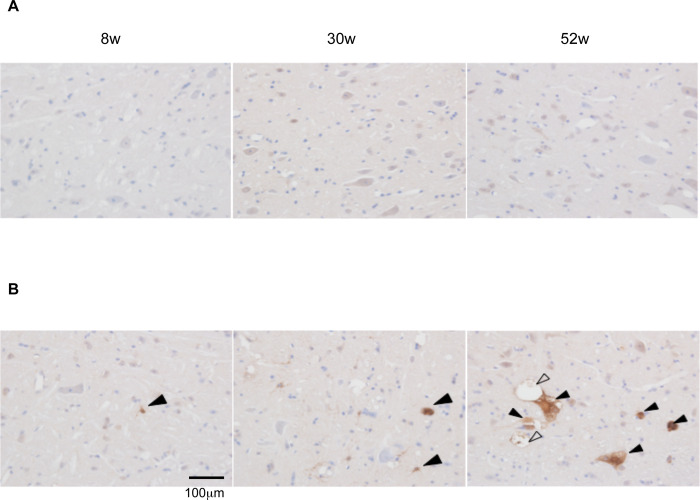
Expression of Serpina3 in mouse brain stem. Representative brain slice images of different ages 8 weeks (left), 30 weeks (middle), 52 weeks (right) of SAMR1(upper panel) and SAMP8 mice (lower panel). (A) SAMR1 (B) SAMP8. In the brain stem of SAMP8, immunoreactivity for Serpina3 (solid arrow) was observed in some astrocytic and neuronal cells of SAMP8, started from 8 weeks followed by 30 and 52 weeks, especially in 52 weeks old SAMP8, while that was not observed in the brain stem of SAMR1. In the brain stem of 52-week-old SAMP8, immunoreactivity for Serpina3 (solid arrow) was observed in some neuronal cells showing vacuolar degeneration (open arrow) (scale bar = 100 μm).

The expression of Serpina3 proteins in SAMP8 and SAMR1 mice brains at various ages (8, 30 and 52 weeks) was examined. The higher signals of Serpina3 proteins were observed at 52 weeks of SAMP8 mice brain stem, colocalized with the vacuolated neuronal structures but were completely absent in case of SAMR1 (Fig 5A and 5B). Representative brain slice images of hippocampus were also shown at different ages of SAMP8 and SAMR1 mice. In comparison with brain stem, few vacuolated structures of Serpina3 proteins were observed at 52 weeks of SAMP8 hippocampus regions but absent in SAMR1 mice (S5A and S5B Fig).

## Discussion

In this paper, we have found polymorphic Serpina3n prolonged the pathogenic oligomeric state of Aβ 42 peptide. Serpina3n is the only member among 14 paralogs of murine a3 Serpins with significant expression in brain under resting condition, becomes the functional human orthologue of SERPINA3 as expressed by astrocytes in the central nervous system [32]. Previous studies identified acute phase response protein, SERPINA3 as a pathological chaperon, accelerated Aβ deposition and colocalized with amyloid beta peptide in AD patient’s brain [33]. Studies also proved that Aβ 42 peptide have structure specific interactions with two β-sheets of SERPINA3 [34, 35]. From the previous hypothetical model of Aβ 42 and SERPINA3 complex, we found both human SERPINA3 I308T (β-sheet 2C chain) and SAMP8 Serpina3n K281R (α helix 8 chain) polymorphisms might be located to the closer proximity of the N terminal end of Aβ 42, the binding site of SERPINA3 (S1D Fig) [35]. Furthermore, we were able to prove that only SAMP8 Serpina3n has the ability to interact with Aβ 42 peptide till 72 hr resulting delayed fibrillization suggesting polymorphism can modulate strong binding efficiency of mutated Serpina3 protein compared to that of JF1 and SAMR1 types (Figs 2C and S3D). So, polymorphisms of mouse and human SERPINA3 probably modulate docking with Aβ 42 and resulted similar effects on the protein structure.

Genetic analysis revealed that human chromosome 14 is related with rapid progression and EOAD or FAD, partly homologous to mouse chromosome 12 [36]. Using age-at-onset (AAO) as a covariate for AD, a linkage signal with a lod score of 1.89 was detected near at 90.7 Mbp (D14S1015) on chromosome 14q32.1, which was within 2 Mbp of *ACT or SERPINA3* gene (at 93.0 Mb) but 19 Mbp distal to the *PRESINILIN 1 (PSEN1)* (at 71.6 Mbp) [37]. In our QTL study, highest lod score 3.54 was observed near at D12Mit133 marker in chromosome 12 LMD locus that was 5.6 Mb close to *Serpina3* (104.4 Mbp) but 25.8 Mb far from *Presinilin 1*(*PS1*) (83.7 Mbp). Even though *PS1* was not on chromosome 12 LMD locus, we did not find any change either in structure or level of expression which negate the possibility that *PS1* to be a candidate gene related with learning and memory dysfunction in SAMP8 mice.

Common signal peptide or promoter region polymorphisms of SERPINA3 were significantly associated with the AD without any influence of apolipoproteinE (APOE) ε4-allele with few inconsistencies [38–41]. It suggests elevated expression of SERPINA3 involves in the pathogenesis of AD. In SAMP8 strain, elevated expression of Serpina3n was observed particularly in the brain stem which may further enhances oligomer retention phenomenon. SERPINA3 was also found to be up-regulated in patients’ brains of schizophrenia and all human prion diseases, signifying the involvement of SERPINA3 in the pathogenesis and progression of prion like fatal neurodegenerative disorders [42, 43]. Another study reported, overexpressed SERPINA3 proteins increased activation of glycogen synthase kinase-3β (GSK-3β), indicating involvement of SERPINA3 induced tau hyperphosphorylation in AD brain [44]. By co-expression of human SERPINA3 with human APP in a transgenic murine model of Alzheimer’s disease, author found increased rate of disease progression indicating human ACT as amyloidogenic cofactor in AD pathogenesis [45].

Mature fibril has long been considered as the cause of disease pathogenesis, however recent evidences suggested oligomeric intermediates formed during fibrillization to be more toxic [22, 23, 46]. Presence of fibrillar plaque in non-demented individuals and elevated levels of soluble oligomeric amyloid beta in AD patients correlate better with the cognitive dysfunction [47–49]. A novel APP mutation (E693delta) in Japanese pedigrees has been suggested as a cause of Alzheimer’s type dementia because of enhanced formation of synaptotoxic Aβ oligomers, resulting inhibited hippocampal long-term potentiation [25]. Plaque bearing transgenic mice, Tg2576 lack neurodegeneration possess intact memory function, concludes no association between plaques and memory loss. In contrast, strong association between oligomeric Aβ*56 and memory loss in Tg2576 mice was observed [50]. Oligomeric Aβ was found to be neurotoxic by activating N-methyl-D-aspartate receptors (NMDARs) induced excess calcium entry into neurons using Tg2576 AD model mice [51, 52].

We found our 0 hr sample of Aβ preparation contains some multimeric forms as shown in S3C Fig and it has been believed such multimeric forms play a role as a seed to accelerate fibrillization causing inconsistent results. Since there is no practical way to keep Aβ peptide in monomeric form and removal of aggregated Aβ peptide is not completely possible using common pretreatment methods [53–55]. Even in the presence of such seeds, polymorphic SAMP8 type Serpina3 or human SERPINA3 I308T has the ability to retain oligomers and prevent fibrillization for more than 24 hr compared to wild type SERPINA3. Thus, the functional differences caused by polymorphic SERPINA3 was found to be resistant against such seed. In addition, dominant effect of polymorphic Serpina3 was also observed by mixing SAMP8 and JF1 types proteins in equimolar ratio (S2B Fig).

Besides, we observed spongy like vacuolated neuronal structures primely in SAMP8 mouse brain stem, may lead its early learning and memory deficits [12]. Additionally, we observed colocalization of Serpina3 with vacuolated structures largely in brain stem and few in hippocampus regions. Our results thus showed polymorphic Serpina3 is related with degenerative neuronal structures in SAMP8 type mouse brain. Amyloid senile plaque was not usually observed in mouse during life span of 2 years, whereas plaque deposition can be found if amyloid-β peptide is overexpressed like in case of triple transgenic AD model mice (3xTg-AD). Furthermore, in the case of SAMP8 mouse retention of considerable proportion of intermediate oligomeric states for delayed time interval may further help to reduce senile plaque formation [12, 56, 57]. Differences in the sites of neurodegeneration between human LOAD patients (hippocampus, cortex) and SAMP8 mice (brain stem), can be explained due to differences in site and level of genes expression. Even though localization of neurodegeneration was different, but mechanism of neuronal cell death might follow a common pathway which will help to study molecular mechanism of oligomeric Aβ induced disease pathogenesis and screen therapeutic agents. SAMP8 can be a very useful rodent model rather being an exact model of LOAD to evaluate impacts of each gene correlated with disease pathogenesis. Besides genome editing of SAMP8 type Serpina3 to that of wild type will help to evaluate the contribution of pathogenic polymorphism towards onset of disease [58].

The heritable component of LOAD still remains elusive may conform to a polygenic model (more alleles of weak genetic effects), and will require larger cohorts to detect smaller genetic effects, offering significant insight into complex disease biology. Since the calculated allelic frequency of human SERPINA3 I308T (rs142398813) in case study was very low (0.006 for AD; 0.014 for DLB), therefore inclusion of large-scale population is required to evaluate the clinical significance of such allele [29, 59].

In conclusion, our findings proved polymorphic SERPINA3 freeze transient toxic state of oligomeric Aβ for longer duration underlying the pathogenic regulatory role of polymorphic SERPINA3 in Aβ pathological studies. Furthermore, it will be helpful to analyze the molecular mechanism of oligomeric Aβ pathogenesis as well as to develop drugs targeting such toxic Aβ forms.

## Materials and methods

### Animals

Four-week-old male SAMP8 (SAMP8/TaSlc) and SAMR1 (SAMR1/TaSlc) mice were purchased from Japan SLC Co. Ltd. (Shizuoka, Japan) and kept in conventional conditions before performing immunohistochemistry experiments.

### Passive avoidance test

Passive avoidance tests were carried out in male and female of SAMP8, JF1, F2 generation mice at 5 months age as described previously [17].

### QTL analysis

QTL analysis was performed as described previously [14]. In this study, we conducted linkage analysis with interval mapping for chromosome 12 by Mapmaker/QTL3.0 b 29 software [www.mapmanager.org]. The position of microsatellite markers was followed according to the UCSC Genome Bioinformatics (http://genome.ucsc.edu/cgi-bin/hgGateway). All animal experiments were carried in accordance with the NIH Guide for the Care of Laboratory Animals and approved by the Animal Care and Use Committee at Hokkaido University.

### RNA sequencing

Hippocampus mRNAs obtained from 2 months of age mice were extracted with trizole method as described previously [60]. RNA-seq analysis was performed using hippocampus mRNA of 2 months age, male mice by Hokkaido System Science (http://www.hssnet.co.jp/index_e.htm). Next generation sequencing (NGS) data was analyzed by using Strand NGS v3.4 software (Strand Life Sciences) and MGSCv37/mm9 was used as reference genome for alignment of read-sequences (https://www.ncbi.nlm.nih.gov/assembly/GCF_000001635.18/). RNA-seq data were analyzed for SAMP8 specific polymorphism such as single nucleotide polymorphism (SNPs), triad base repetition, unique alternative splicing events and long intervening non-coding RNAs. We have deposited RNA-seq data deposition in GEO data repository site (Accession number: PRJNA693683).

### Micro array analysis

Micro array analysis was performed using Agilent SurePrint G3 Mouse Gene Expression 8X60K array using total RNAs of 5 months age of both male and female mice of JF1, SAMR1 and SAMP8 strains through Takara Bio (http://www.clontech.com/takara). We have analyzed and compared the expression level of genes present in chromosome 12 LMD locus using microarray data of 3 mice at 5 months of age from each strain. Microarray data was deposited in GEO data repository site (https://www.ncbi.nlm.nih.gov/geo/query/acc.cgi?acc=GSE164731).

### Construction of mouse *Serpina3*

Full length *Serpina3n* gene of SAMP8, SAMR1 and JF1types were amplified by PCR (Bio-Rad, USA) using hippocampus cDNA as templates with primers 5′-TCACGGTACCTGGCAGCTGGCTGGTTTCAGCTCTGT-3′ and 5′-GTTCGGATCCCCTTTGGGGTTGGCTATCTTGGCTATAA-3′. PCR was conducted according to following condition: 95°C 3 minutes, (for denaturation 95°C 30 sec, annealing 54°C 30 seconds, extension period 72°C 90 seconds) 5 cycles followed by (for denaturation 95°C 30 sec, annealing 60°C 30 seconds, extension period 72°C 90 seconds) 40 cycles, 72°C 3 minutes and 24°C 1 minutes. The amplified genes were inserted into pBluescript II SK (-) vector (Stratagene, USA) and sequenced. The sequence was obtained by ABI Sequencher and results were compared using Sequencher ver. 5.4.6. (www.genecodes.com). The amplified fragments of *Serpina3n* genes were then inserted into mammalian expression vector pEF1/Myc-His C (Invitrogen, USA) respectively. Plasmids were prepared by high purity midi prep plasmid purification kit (Marligen Bioscience, USA) and suspended in ultrapure water.

### Human *SERPINA3* mutagenizaion

Full-length human *SERPINA3* gene was synthesized and cloned into pUC57 vector by GenScript. The human *SERPINA3* gene (wild type) was inserted into mammalian expression vector pEF1/Myc-His C (Invitrogen, USA). Then this control construct was mutagenized to carry Isoleucine 308 Threonine and using 5′-TTCAGAGAGACAGGTGAGCTCTACCTGCCA-3′ & 3′-CTGTCTCTCTGAACTCCAG AGAGTCTCTC-5′ primers combination. PCR condition was performed following, 95°C 3 minutes, (for denaturation 95°C 30 sec, annealing 52°C 30 seconds, extension period 72°C 6 minutes) 5 cycles followed by (for denaturation 95°C 30 sec, annealing 60°C 30 seconds, extension period 72°C 6 minutes) 40 cycles, 72°C 3 minutes and 24°C 1 minutes. Plasmids were prepared by high purity midi prep plasmid purification kit (Marligen Bioscience, USA) and suspended in ultrapure water. From the database, we got the reference sequence number for *SERPINA3 I308T* as rs142398813 for this missense human variant (https://www.ncbi.nlm.nih.gov/snp/rs142398813).

### Expression of recombinant SERPINA3 proteins

Recombinant SERPINA3 proteins (both mouse and human types) were expressed in Expi293F cells using Expi293F expression system kit of Gibco (Life Technologies, Japan) according to the manufacturer standard protocol. Briefly, 3 to 4 days of post transfection, Expi293F expression medium was collected, mixed with Ni-NTA resins (Qiagen, Germany) to bind with polyhistidine-tagged SERPINA3 proteins. Finally, recombinant proteins were eluted using Ni-NTA beads elution buffer NPI-250 prepared according to the Qiagen supplementary protocol (www.qiagen.com). After elution of expressed recombinant SERPINA3 proteins, eluting buffer containing 500 mM imidazole was exchanged with PBS buffer (pH 7.4) using Amicon Ultra-0.5 30K filter device (Millipore, Ireland). Purified recombinant mouse and human SERPINA3 proteins were centrifuged 22000 rpm for 90 minutes at 4°C to precipitate insoluble particles. Purified proteins were then collected without touching bottom of the ependorffs and stored at -80°C till use. The recombinant proteins were run into 10% SDS-PAGE and stained with Oriole fluorescent gel stain solution (Bio-Rad, USA) according to manufacturer`s protocol. Molecular sizes were determined using size marker in SDS-PAGE like Precision Plus Protein unstained standard (Bio-Rad, USA). The expected size of recombinant proteins was for mouse 47 kDa and human 48 kDa whereas we got higher size of smear bands around 50 to 65 kDa for mouse (lanes 2–4) and around 50 to 80 kDa in case of human SERPINA3 proteins (lanes 5–6) may be due to different glycosylation patterns (S1E Fig). The amount of protein was quantified using ImageJ 1.46r software (http://imagej.nih.gov/ij).

### SAMP8 Serpina3 protein inactivation protocol

Inactivation of SAMP8 Serpina3 protein was performed according to standard protocol with some modification [61]. Briefly, lyophilized trypsin gold powder (Promega, USA) was reconstituted to make final 1 μg / μl in 50 mM acetic acid solution. Trypsin solution was diluted to make final 36 μM solution using 0.05 M Tris-Cl solution (pH 8.0). Substrate of trypsin Na-Benzyol-DL-arginine-4-nitroanilide hydrochloride (Sigma, USA) was dissolved in 90% DMSO to make final 10 mM solution. Recombinant SAMP8 Serpina3 protein was at first heat inactivated at 95°C for 30 minutes, then recombinant protein was proteolytically cleaved by mixing with trypsin at optimum molar ratio of Serpina3: trypsin (16: 1) and incubated at 37°C for overnight followed by heat inactivation again 95°C for 30 minutes. Then this heat inactivated and proteolytically cleaved SAMP8 Serpina3 protein was used in experiments.

### Preparation of amyloid beta 42

Human amyloid beta (Aβ) 42 peptide was purchased from Peptide Institute Inc (Japan), already treated with trifluoroacetic acid and lyophilized powder form before use as described previously [35, 54, 55]. We kept Aβ 42 peptide at r.t. for 30 minutes and then dissolved using filtered 0.1% NH3 solution (Sigma-Aldrich, USA) according to manufacturer`s instruction. The preparation was ultra-centrifuged using TL-100 ultracentrifuge incorporating TLS 55 rotator (Beckman, USA) at 4°C, 55000 rpm for 3 hr. After ultra-centrifugation, supernatant was collected without touching bottom of polycarbonate centrifuge tubes (Beckman, USA), aliquoted into micro-tubes and quantified by Bradford protein assay method [62]. Aliquoted tubes were immediately dipped into liquid N_2_ and finally stored at—80°C as stock solution up to one month. During experiments frozen samples were thawed and diluted with buffer solution (PBS, pH 7.4). Thawed samples were consumed in one experiment and repetitions of freeze-thaw cycles were strictly avoided.

### Oligomerization assay protocol

Freshly prepared Aβ 42 peptide was then diluted with PBS (pH 7.4) to make final 45 μM concentration and then incubated at 25°C without agitation using Bio-Shaker M.BR-022UP (Taitec, Japan) in the absence or presence of 2.25 μM (final concentration) of different sources of SERPINA3 proteins from mouse and human species. During incubation 4–5 μl assay sample was aliquoted at different time intervals (0, 4, 8, 12, 24, 36, 48 and 72 hrs).

### Transmission Electron Microscope (TEM) observation

For TEM observation experiment, we preincubated samples at 0, 8, 24 and 72 hrs. Sample seed was prepared according to standard protocol [63]. Briefly, 1 μl incubated sample was mixed with 2 μl assay buffer (PBS, pH 7.4) and put onto Bemis Parafilm M (Sigma, USA). Then 200 mesh carbon-coated copper grid (Nisshin EM Co. Ltd., Tokyo) was placed invertly onto sample mixture and allowed to adsorb for 10 minutes. Excess sample was removed from the grid using kimwipe paper. The grid was negatively stained using 3 μl, 2% phosphotungstic acid (pH 7.0) for 3 minutes and excess staining solution was removed using kimwipe paper. The negatively stained sample grid was then washed with the staining solution at 10 sec intervals for twice. Finally, excess solution of sample grid was removed same as above and allowed for air dried at room temperature for couple of minutes. The sample grids were kept at desiccator for storage till observation under electron microscope. Before sample loading carbon coated copper grids were hydrophilized using Eiko Ion coater IB-3 (Tokyo, Japan). Samples were observed with an excitation voltage of 80 kV using JEM-1400TC TEM (JEOL, Tokyo, Japan). Each time point for each condition was triplicated to obtain statistical significance (One-way ANOVA with post-hoc Tukey HSD).

Prevalence frequencies were calculated by tallying various physical states of Aβ 42 peptide. Briefly, each TEM seed was divided into four observation quarters (A, B, C, D) under TEM as nano-space map. We took images from every quarters randomly at once for each seed. Then counted the number of observation fields for each physical state, tabulated, quantified and expressed as percent prevalence. We choose 500 nm scale as standard to calculate. Different physical states of Aβ peptide were compared as described previously [20, 22, 23].

### Thioflavin T (ThT) fluorescence assay

Aβ 42 fibrillization was assessed by Thioflavin T (ThT) (Sigma, USA) assay as described previously [64]. ThT stock solution (10 mM) was prepared using 100% ethanol and 50 mM glycine NaOH buffer (pH 8.5) was used to make working solution (300 μM). Aβ 42 was mixed in the presence or absence of recombinant SERPINA3 proteins in a molar ratio of 50: 1 for Aβ 42: SERPINA3 (10 μM: 0.2 μM). In experimental procedure, final 10 μM ThT solution in PBS buffer (pH 7.4) was used as assay buffer at r.t. without any agitation. The fluorescence intensity of 10 μM ThT solution in 50 mM glycine NaOH and PBS buffers were used as background signal. We mixed SAMP8 and JF1 types Serpina3 proteins in equimolar ratio (1:1), and performed ThT assay to check effect of polymorphism on Aβ 42 peptide fibrillization process.

Cell carrier-96 clear bottom Perkin Elmer plate (Perkin Elmer, USA) was used to observe the conformational changes of Aβ peptide 42 in the presence or absence of recombinant SERPINA3 proteins by mixing with ThT solution under high-content imaging system Operetta CLS (Perkin Elmer, USA). Cerulean special channel (excitation spectrum 410–430 nm and emission spectrum 460–500 nm) was used to assess fibrillization process of Aβ 42 peptide. Harmony software (version 3.5.1) was used to capture images and analyze the thioflavin T signal intensities of fibrillization process at various time course events. Fluorescent intensity was calculated by summation of all the observation fields per well captured at minimum threshold value of 0.4. Standard error of mean (S.E.M) was calculated from triplicated samples.

### Western blot assay

To identify various low molecular weight (LMW) Aβ 42 peptide conformations in absence or presence or SERPIINA3 proteins, we carried out Western blot assays using native PAGE as described previously [20]. Briefly, 5 μl of preincubated samples were mixed with 3x native sample buffer then applied onto 6–20% (w/v) Tris-glycine poly acryl amide gradient gel, electrophoresed and transferred onto pure Nitrocellulose transfer membrane (MSI, USA). After blocking with 4% skimmed milk in PBST at r. t. for 1 hr, membranes were probed with 6E10 (1: 5000), mouse monoclonal IgG (BioLegend, USA) antibody and kept at 4°C overnight. IRDye 800CW goat (polyclonal) anti-mouse IgG (H+L) (LI-COR, USA) was used as secondary antibody (x15000) to detect signals with LI-COR Odyssey image studio version 5.2 (http://www.licor.com/bio) according to manufacturer’s instructions. Similar way to perform SDS-PAGE, preincubated samples were mixed with 3x sample buffer in absence of reducing agent (beta mercapto ethanol). Without heat denaturation, samples were then applied onto 6–20% (w/v) Tris-glycine SDS-PAGE gradient gel. After primary antibody (6E10) treatment, HRP-conjugated goat anti-mouse IgG (H+L chain) (MBL, Japan) secondary antibody (1: 2500) was used to detect signals. Bands were visualized using Amersham ECL prime Western blotting detection reagent (GE, USA). Molecular sizes were determined using size markers in SDS-PAGE like Precision Plus Protein standards markers, Dual color (Bio-Rad, USA). As a reference in native PAGE Dual Color Xtra (most left lane) (Bio-Rad, USA) and All blue (most right lane) (Bio-Rad, USA) were used. These experiments were repeated three times to confirm reproducibility of detection.

### Pull-down assay

To examine interactions between mouse Serpina3n recombinant proteins and Aβ 42 peptide, pull-down assay was performed as previously described with few modifications [65]. HFIP treated biotinoyl-beta-amyloid (1–42) was purchased from JPT Peptide Technologies (Germany). Briefly, 45 μM Aβ 42 (0% biotinylated Aβ 42), mixture of Aβ 42 and biotinylated Aβ 42 (36 μM: 9 μM) (20% biotinylated Aβ 42) were prepared. Then 20 μl of Aβ 42 mixtures were preincubated with mouse Serpina3n (2.25 μM) for 24, 48 and 72 hrs as described above (see Oligomerization assay protocol). High performance magnetic nanoparticles of streptavidin beads (Tamagawa, Japan) were washed and blocked with 2% BSA in PBS solution (beads mixture) at 4°C for 2 hr. 10 μl of preincubated sample of each condition was stirred with 90 μl streptavidin beads mixture at 4°C for 1 hr and then pull-down using a magnetic ependorffs stand. The pulled down streptavidin samples were washed, suspended into 20 μl of PBS, then divided equally for dot blot and Western blot assay using 10% SDS-PAGE. Mouse Serpina3n was detected using anti c-myc mouse monoclonal antibody (9E10: sc-40, Santa Cruz, USA) with 1000-fold dilution. IRDye 800CW goat (polyclonal) anti-mouse IgG was used as secondary antibody to detect both Serpina3 and Aβ 42 as described above (Western blot assay).

### Cell culture

SH-SY5Y neuroblastoma cell lines were purchased from ATCC (USA). Cells were maintained in medium prepared with DMEM, Ham’s F-12, 2 mM l-glutamine and 10% FBS and incubated at 37°C in 5% CO2. The medium was changed every 2–3 days and cells were split using 0.2% Trypsin PBS EDTA preparation. To neutralize trypsin activity, culture medium was used before cell seeding.

### Cytotoxicity assay

To conduct cytotoxicity assay, SH-SY5Y cells were cultured in medium prepared with DMEM, Ham’s F-12, 2 mM l-glutamine and 1% FBS. Cells were plated in cell carrier-96 clear bottom Perkin Elmer (USA) plates with approximately 2 x 10^4^ cells / well in 100 μl. After 48 hr of cell seeding 0, 12, 24, 36 and 48 hours preincubated Aβ 42 peptide samples such as Aβ 42 alone or with SERPINA3 proteins were mixed with new culture medium containing final 10 ug/mL levofloxacin hemihydrates (Wako, Japan) and added into properly labeled plates. The molar ratio of Aβ 42: SERPINA3 was maintained as final 10 μM: 0.5 μM (20: 1) in assay condition. After another 48 hr of exposure, cytotoxicity effects were determined through LDH assay (LDH-Glo kit, Promega, USA), membrane permeability assay with Propidium Iodide (PI) (Sigma, USA), morphological changes under bright field microscope, Hoechst 33342 (Calbiochem, USA), or DAPI (Sigma, USA) staining to monitor intactness of nucleus. Axonal integrity of microtubules (major component of cell cytoskeleton) was confirmed using anti beta III tubulin antibody (Abcam, USA).

### Immunostaining of Serpina3 in mouse brain

After treatment with hydrogen peroxide and blocking with 2% bovine serum albumin in PBS including 0.05% Tween 20 (PBST) for 30 min, the sections were incubated with goat anti mouse SerpinA3N antibody (x400, R&D system) in PBST at 4°C overnight. The sections were washed with PBS and then incubated with a polymer solution conjugated with anti-goat IgG antibody and horseradish peroxidase (HRP) (Histofine Simple Stain MAX PO (G), Nichirei Biosciences Inc., Tokyo, Japan) and developed with 3, 3’- di amino benzidine tetra hydrochloride (DAB) at r.t. for 5–7 minutes. The sections were counter stained with hematoxylin.

### Statistical analysis

All data were expressed as means ± SEMs. The statistical significance was analyzed using JMP Pro 14.2.0 (64-bit) (http://www.jmp.com) followed by Pearson’s chi-squared test, One-way ANOVA with Tukey and Kramer’s honestly significance difference test. Graphs were processed using Prism 8.0 (Graph Pad Software).

## Supporting information

S1 Fig Expression data of Serpina3. (A) Expression profile of homologous Serpina present in chromosome 12 LMD locus. (Data represent Mean ± SEM, n = 3). Graph was created using combined microarray data of male (n = 1) and female (n = 2) 5M age mice of SAMP8, SAMR1 and JF1 strains. Among the homologous genes, only Serpina3n dominantly expressed. (B) Expression profile of homologous *Serpina* gene present in chromosome 12 LMD locus. Graph was prepared using 2M age male mice (n = 1) of each strain. FPKM = Fragments Per Kilobase of exon per Million reads mapped (C) High throughput sequencing results obtained by Strand NGS v3.4 software showing one synonymous (S271S) and two non-synonymous polymorphisms (M273L, K281R) specific to SAMP8 type Serpina3n. (D) Positions of SAMP8 specific Serpina3n SNPs and human SERPINA3 I308T were shown in the 3D structure. (E) Representative image of EXPI293F expressed recombinant SERPINA3 proteins in 10% SDS-PAGE. Recombinant proteins were stained with Oriole fluorescent gel stain and molecular size marker was shown in lane 1. Mouse Serpina3 recombinant proteins were represented in lane 2 = JF1, lane 3 = SAMR1 and lane 4 = SAMP8, showing smear of bands around 50–65 kDa. Human SERPINA3 recombinant proteins were represented in lane 5 = SERPINA3 WT and lane 6 = SERPINA3 I308T, showing smear of bands ranges from 50–80 kDa.
(TIF)

S2 Fig Graphical presentation of thioflavin T fluorescence assay of mouse Serpina3 proteins. (A) Comparison of polymorphic active & inactive SAMP8 type Serpina3 on Aβ 42 peptide fibrillization. (B) Effect of equimolar mixture of JF1 and SAMP8 type Serpina3 proteins on Aβ 42 peptide fibrillization. Data represent Mean ± SEM, n = 3. Here, AFU refers arbitrary fluorescence units.
(TIF)

S3 Fig Conformational differences of Aβ 42 peptide in presence of SAMP8 type Serpina3. Representative images of Aβ 42 peptide in absence or presence of mouse Serpina3 recombinant proteins in Western blot assay using 6% to 20% gradient native gel (A) 0 to 18 hrs and (B) 24 to 48 hrs preincubated samples. (C) SDS-PAGE image of 0 hr preincubated samples. Open triangle showed presence of tetramer (~18 kDa) and trimer (~13.5 kDa) Aβ peptide conformations. (D) Representative image of SAMP8 type Serpina3 using pull-down assay at 72 hr to compare interactions between mouse Serpina3 recombinant proteins with Aβ 42 peptide using 10% SDS-PAGE. Here, input indicates samples before pull-down assay, whereas pulldown refers samples collected after streptavidin beads were precipitated using magnet. Only SAMP8 Serpina3 can be detected due to coprecipitation with Aβ 42 peptide in presence of biotinyl 20% Aβ 42 at 72 hr.
(TIF)

S4 Fig LDH assay of toxic Aβ 42 induced cell death using SHSY5Y neuroblastoma cell line. Bar graphs showing luminescence value (AFU) in y-axis at different preincubated time points indicated with thick vertical line in x-axis. (A) Aβ 42 alone or mixed with mouse polymorphic Serpina3n. Significance levels were tested SAMP8 vs. SAMR1 & SAMP8 vs. JF1. (B) Aβ 42 alone or mixed with human polymorphic SERPINA3. Significance levels were tested against SERPINA3 I308T vs. SERPINA3 wild. Data represent Mean ± SEM. (n = 3, * *P* ≤ 0.05; ** *P* ≤ 0.01. Tukey and Kramer’s honestly significance difference test was used). Representative images of most delayed time points showing degenerated and intact microtubules stained with anti tubulin beta III (green color) (n = 3). (C) 36h preincubated sample of Aβ 42 alone or mixed with mouse polymorphic Serpina3. (D) 48h preincubated sample of Aβ 42 alone or mixed with human polymorphic SERPINA3. Control samples (Cell culture medium, Cell culture medium + PBS) (n = 3), (E) Hoechst 33342 showing blue signal of intact nucleus; Propidium iodide (PI) identified red signal of damaged cell DNA. (F) DAPI, blue dye for nucleus & anti-tubulin beta-III, green dye (with Alex488) for micro tubular structures.
(TIF)

S5 Fig Expression of SerpinA3 in mouse brain hippocampus. Representative brain slice images of different ages 8 weeks (left), 30 weeks (middle), 52 weeks (right) of SAMR1(upper panel) and SAMP8 mice (lower panel). (A) SAMR1 (B) SAMP8. In the hippocampus of SAMR1 and SAMP8, immunoreactivity for Serpina3 was rarely observed, except for 52 weeks old SAMP8, in which a few vacuolated structures (arrow) were stained with the antibody (Scale bar = 100 μm). (C-D) Magnified view of 52 weeks SAMP8 hippocampal vacuolated regions.
(TIF)

S1 Table Position of polymorphism of JF1, SAMR1 and SAMP8 type Serpina3n proteins in the open reading frame (ORF). (PDF)

S1 Raw images (PDF)
