# Supplementary figures and images for: Polymorphic SERPINA3 prolongs oligomeric state of amyloid beta
Source: PLoS One. 2021 Mar 4;16(3):e0248027. doi: 10.1371/journal.pone.0248027 (PMC7932536; doi:10.1371/journal.pone.0248027)

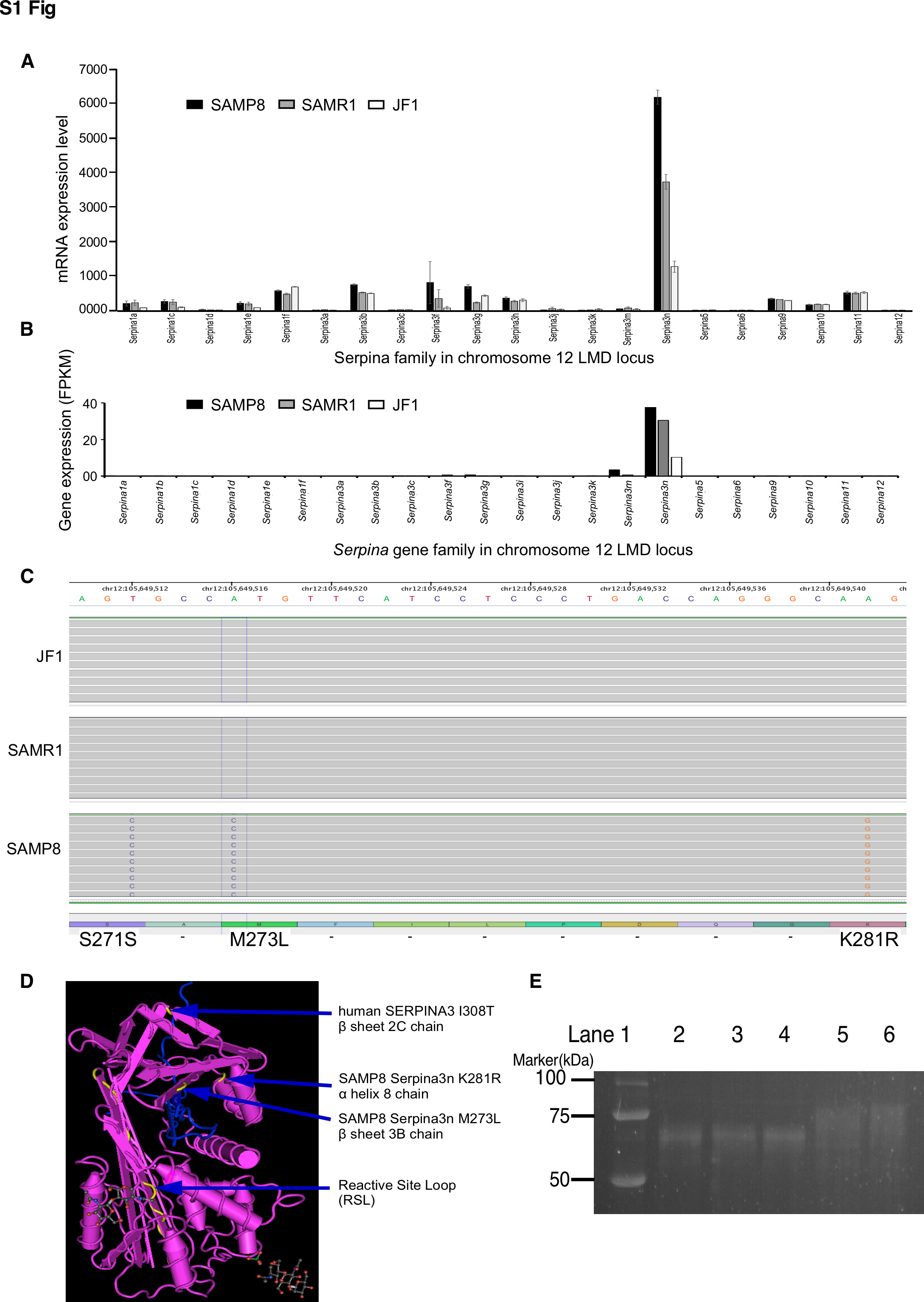

Supplement: S1 Fig — (A) Expression profile of homologous Serpina present in chromosome 12 LMD locus. (Data represent Mean ± SEM, n = 3). Graph was created using combined microarray data of male (n = 1) and female (n = 2) 5M age mice of SAMP8, SAMR1 and JF1 strains. Among the homologous genes, only Serpina3n dominantly expressed. (B) Expression profile of homologous Serpina gene present in chromosome 12 LMD locus. Graph was prepared using 2M age male mice (n = 1) of each strain. FPKM = Fragments Per Kilobase of exon per Million reads mapped (C) High throughput sequencing results obtained by Strand NGS v3.4 software showing one synonymous (S271S) and two non-synonymous polymorphisms (M273L, K281R) specific to SAMP8 type Serpina3n. (D) Positions of SAMP8 specific Serpina3n SNPs and human SERPINA3 I308T were shown in the 3D structure. (E) Representative image of EXPI293F expressed recombinant SERPINA3 proteins in 10% SDS-PAGE. Recombinant proteins were stained with Oriole fluorescent gel stain and molecular size marker was shown in lane 1. Mouse Serpina3 recombinant proteins were represented in lane 2 = JF1, lane 3 = SAMR1 and lane 4 = SAMP8, showing smear of bands around 50–65 kDa. Human SERPINA3 recombinant proteins were represented in lane 5 = SERPINA3 WT and lane 6 = SERPINA3 I308T, showing smear of bands ranges from 50–80 kDa. (TIF) [file pone.0248027.s001.tif]

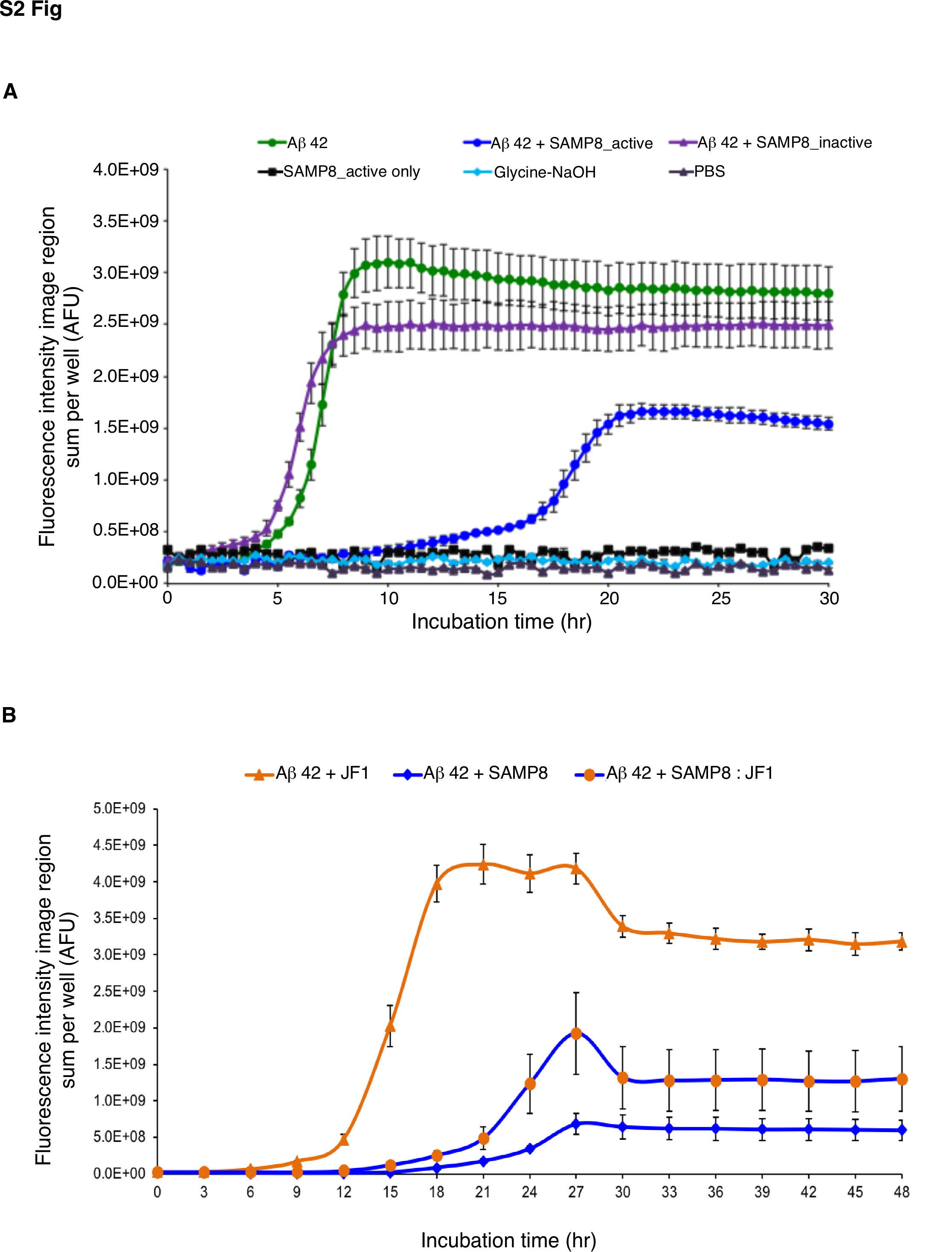

Supplement: S2 Fig — (A) Comparison of polymorphic active & inactive SAMP8 type Serpina3 on Aβ 42 peptide fibrillization. (B) Effect of equimolar mixture of JF1 and SAMP8 type Serpina3 proteins on Aβ 42 peptide fibrillization. Data represent Mean ± SEM, n = 3. Here, AFU refers arbitrary fluorescence units. (TIF) [file pone.0248027.s002.tif]

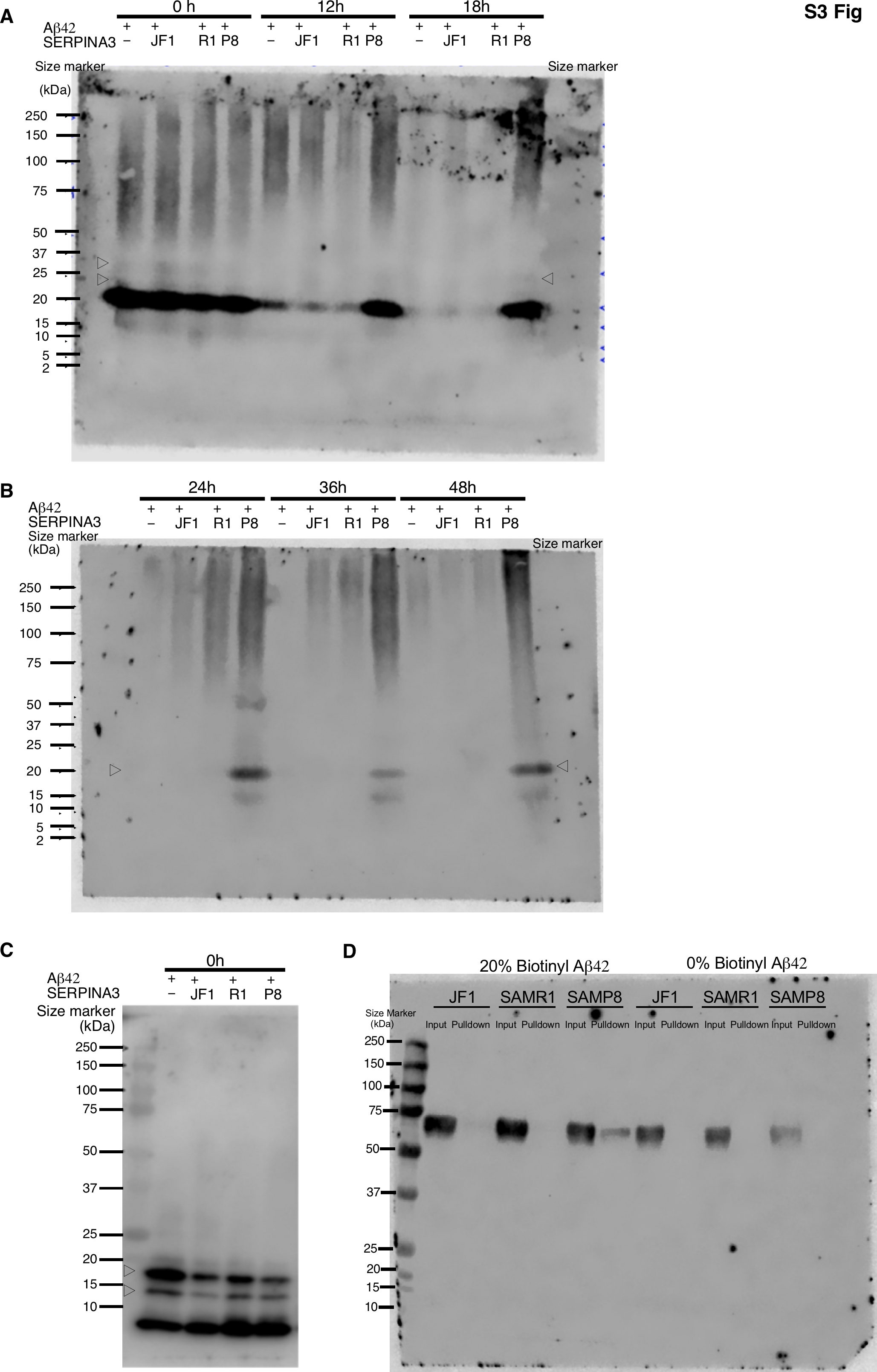

Supplement: S3 Fig — Representative images of Aβ 42 peptide in absence or presence of mouse Serpina3 recombinant proteins in Western blot assay using 6% to 20% gradient native gel (A) 0 to 18 hrs and (B) 24 to 48 hrs preincubated samples. (C) SDS-PAGE image of 0 hr preincubated samples. Open triangle showed presence of tetramer (~18 kDa) and trimer (~13.5 kDa) Aβ peptide conformations. (D) Representative image of SAMP8 type Serpina3 using pull-down assay at 72 hr to compare interactions between mouse Serpina3 recombinant proteins with Aβ 42 peptide using 10% SDS-PAGE. Here, input indicates samples before pull-down assay, whereas pulldown refers samples collected after streptavidin beads were precipitated using magnet. Only SAMP8 Serpina3 can be detected due to coprecipitation with Aβ 42 peptide in presence of biotinyl 20% Aβ 42 at 72 hr. (TIF) [file pone.0248027.s003.tif]

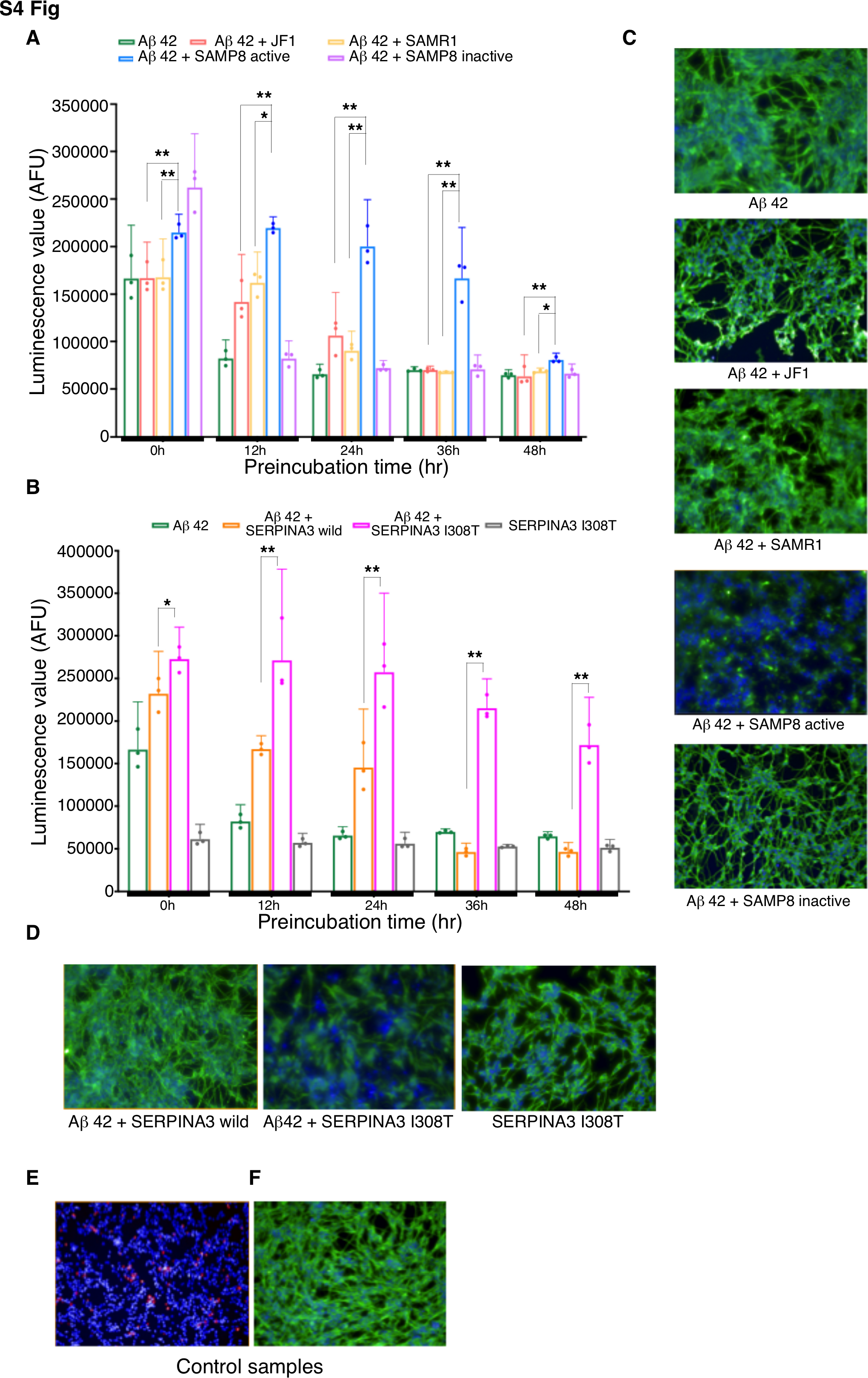

Supplement: S4 Fig — Bar graphs showing luminescence value (AFU) in y-axis at different preincubated time points indicated with thick vertical line in x-axis. (A) Aβ 42 alone or mixed with mouse polymorphic Serpina3n. Significance levels were tested SAMP8 vs. SAMR1 & SAMP8 vs. JF1. (B) Aβ 42 alone or mixed with human polymorphic SERPINA3. Significance levels were tested against SERPINA3 I308T vs. SERPINA3 wild. Data represent Mean ± SEM. (n = 3, * P ≤ 0.05; ** P ≤ 0.01. Tukey and Kramer’s honestly significance difference test was used). Representative images of most delayed time points showing degenerated and intact microtubules stained with anti tubulin beta III (green color) (n = 3). (C) 36h preincubated sample of Aβ 42 alone or mixed with mouse polymorphic Serpina3. (D) 48h preincubated sample of Aβ 42 alone or mixed with human polymorphic SERPINA3. Control samples (Cell culture medium, Cell culture medium + PBS) (n = 3), (E) Hoechst 33342 showing blue signal of intact nucleus; Propidium iodide (PI) identified red signal of damaged cell DNA. (F) DAPI, blue dye for nucleus & anti-tubulin beta-III, green dye (with Alex488) for micro tubular structures. (TIF) [file pone.0248027.s004.tif]

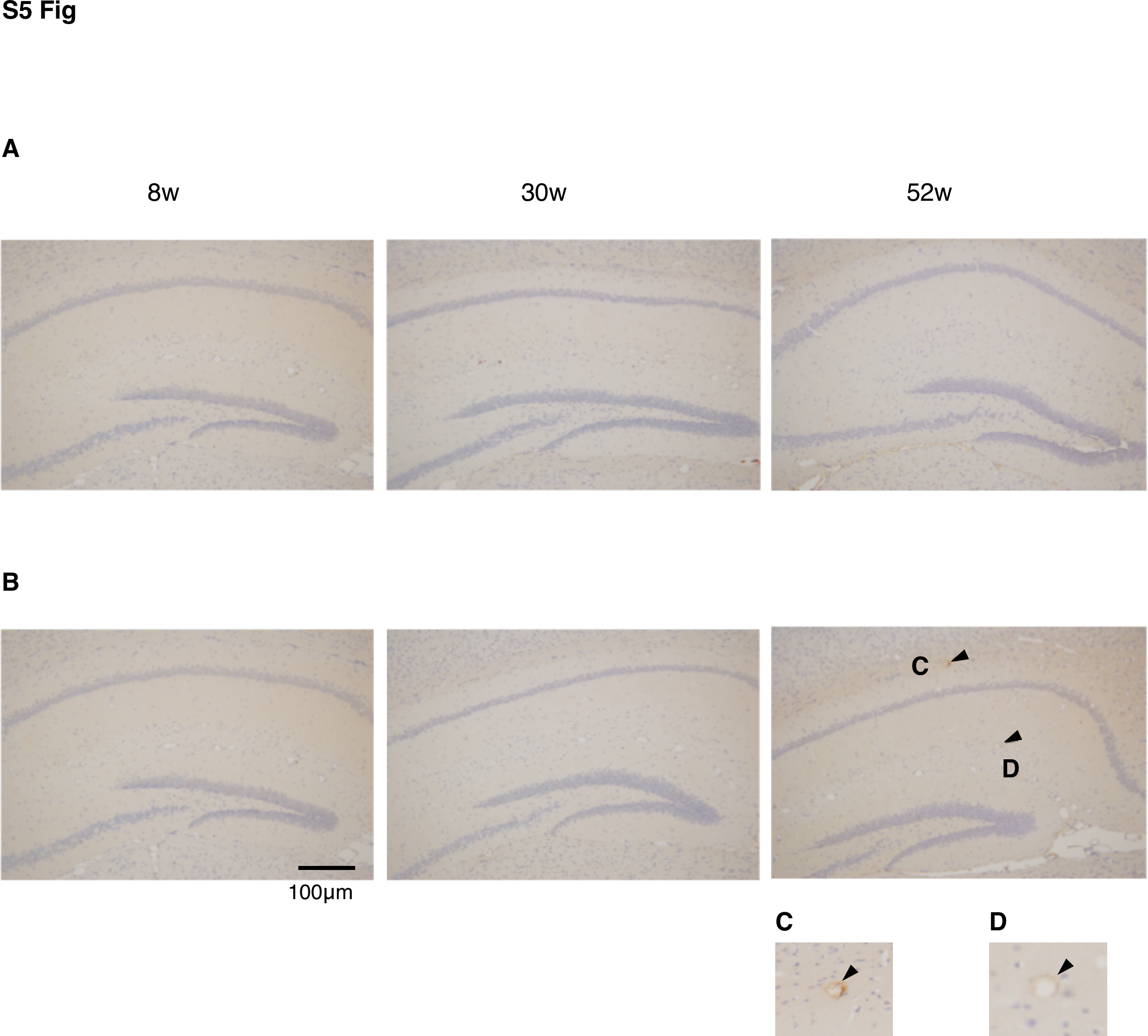

Supplement: S5 Fig — Representative brain slice images of different ages 8 weeks (left), 30 weeks (middle), 52 weeks (right) of SAMR1(upper panel) and SAMP8 mice (lower panel). (A) SAMR1 (B) SAMP8. In the hippocampus of SAMR1 and SAMP8, immunoreactivity for Serpina3 was rarely observed, except for 52 weeks old SAMP8, in which a few vacuolated structures (arrow) were stained with the antibody (Scale bar = 100 μm). (C-D) Magnified view of 52 weeks SAMP8 hippocampal vacuolated regions. (TIF) [file pone.0248027.s005.tif]

Fig 3\_E\_raw image

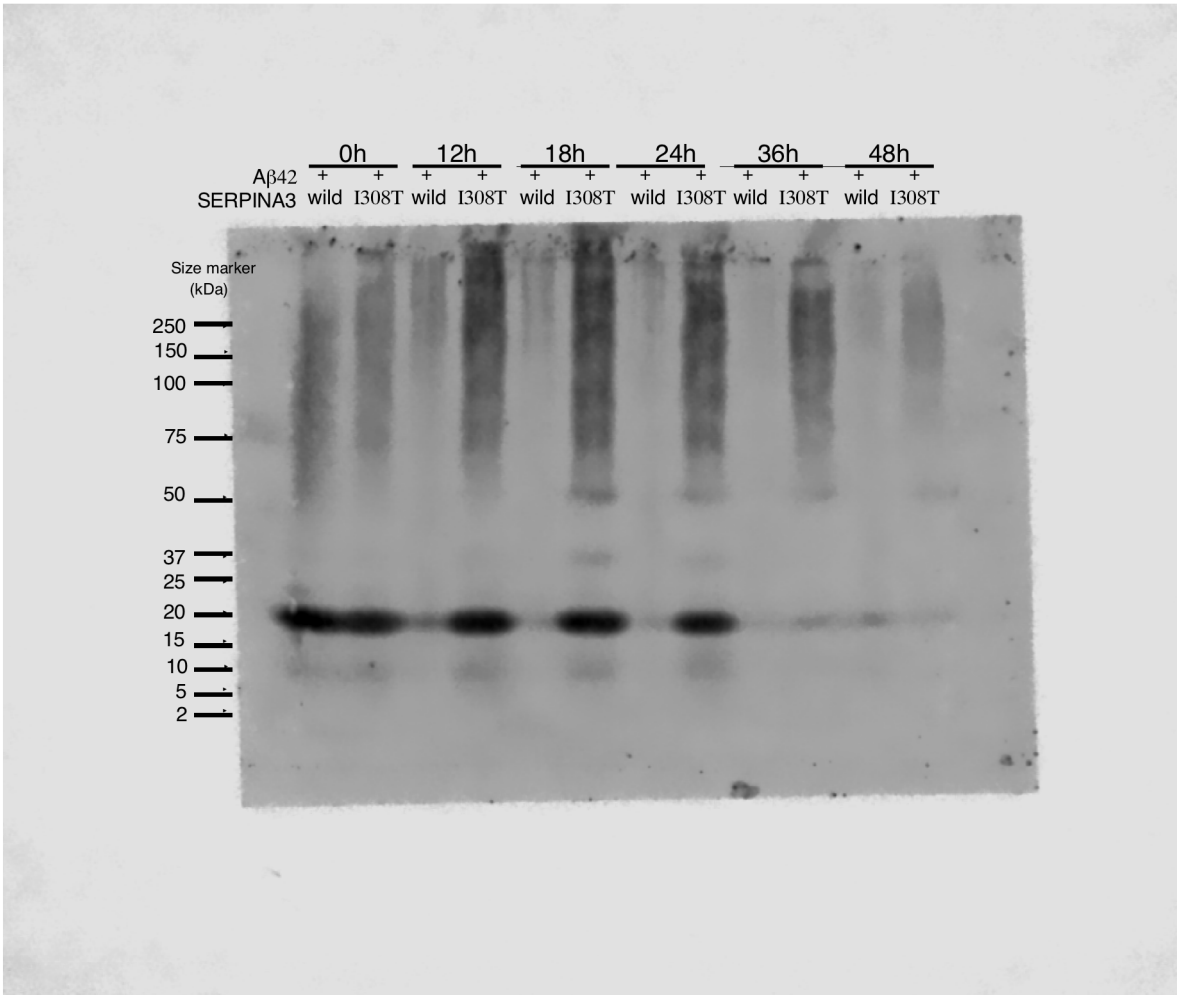

# S1\_D\_Fig\_raw\_images

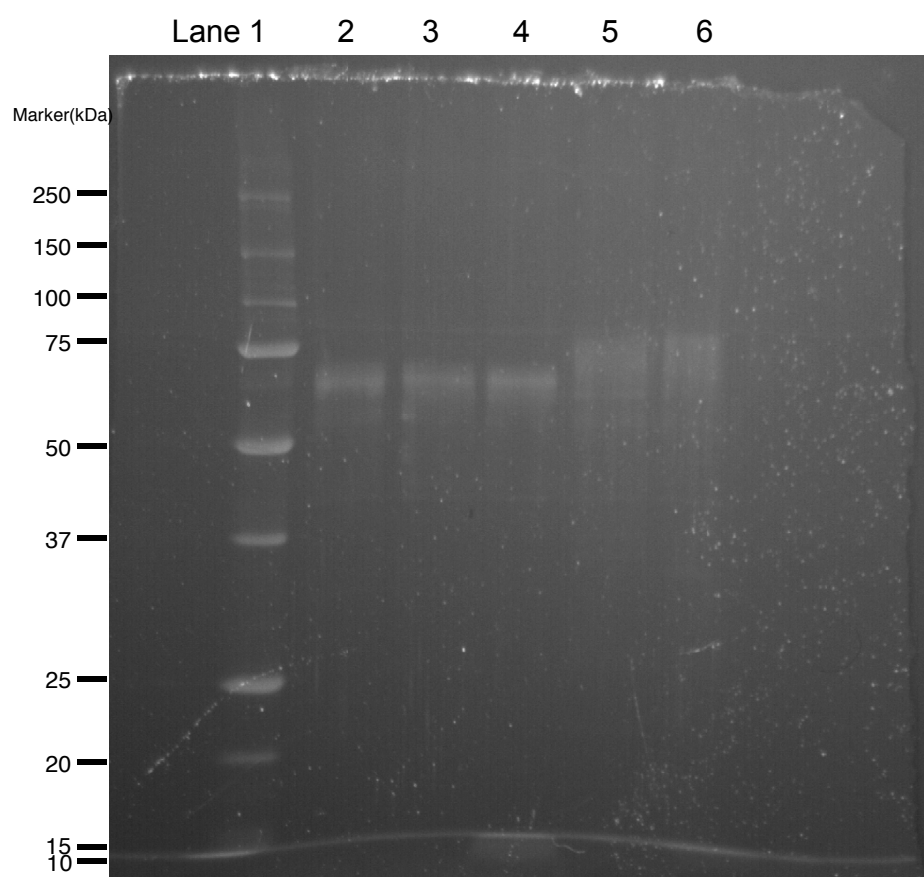

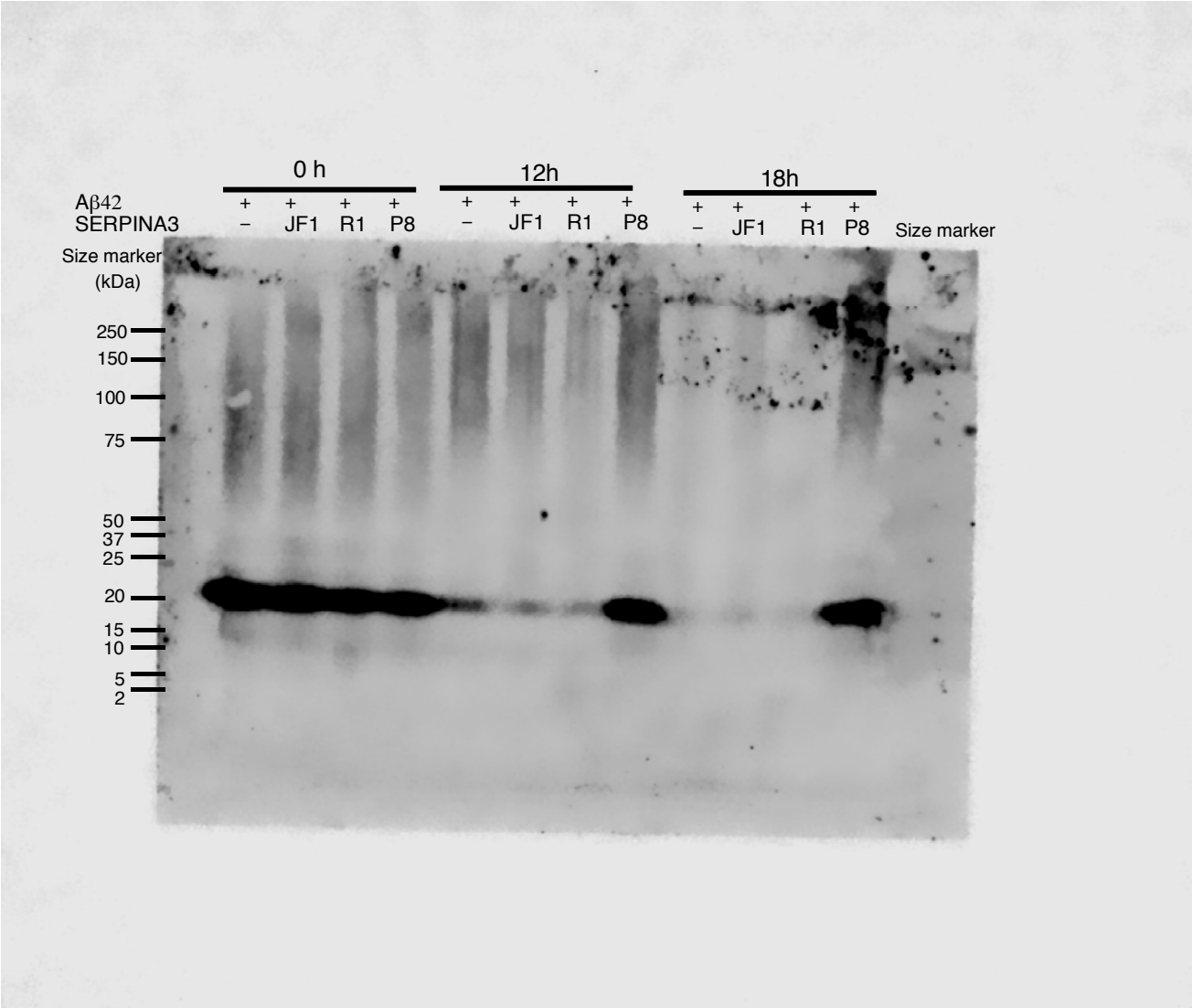

S3\_B\_Fig\_raw\_images

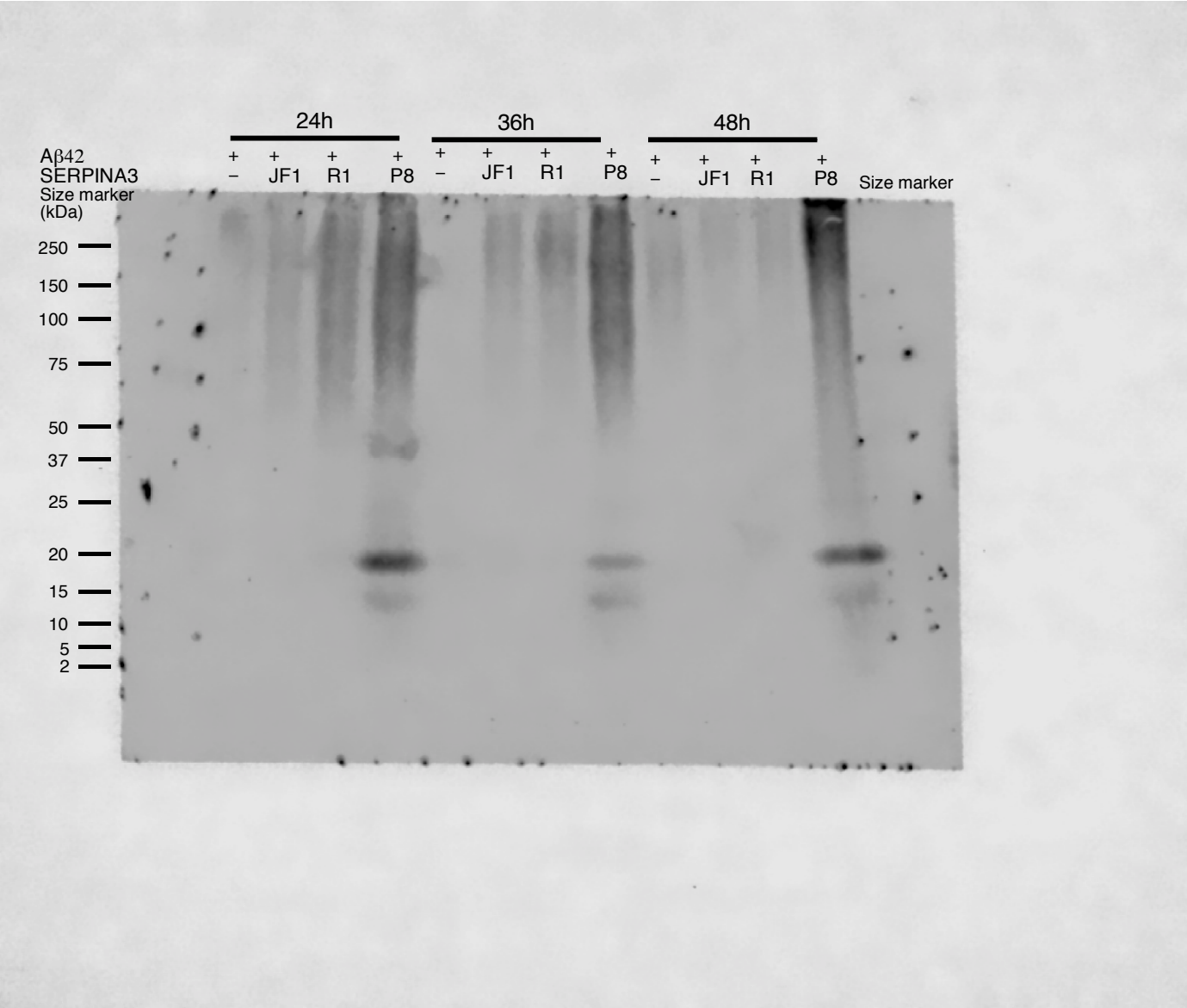

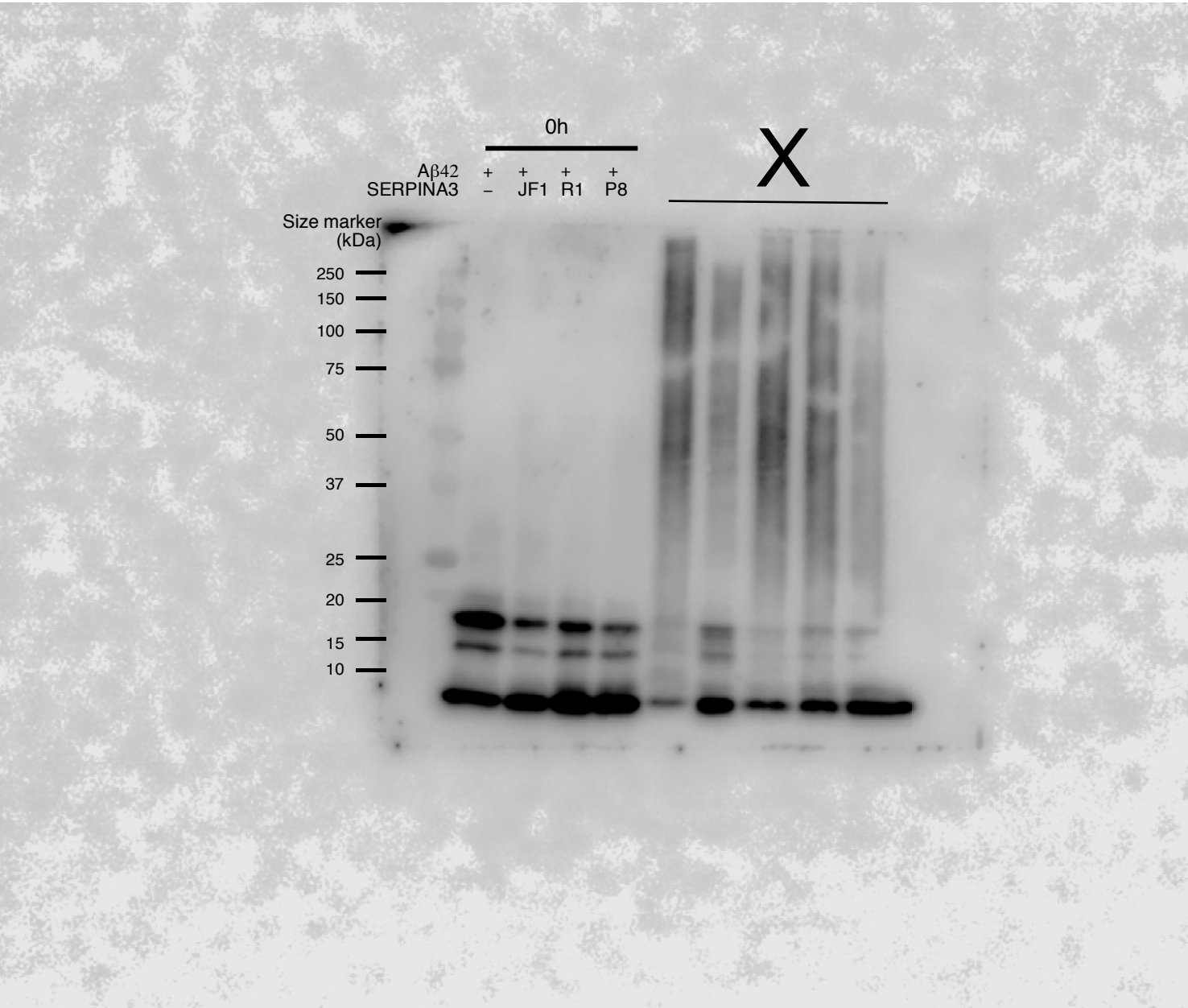

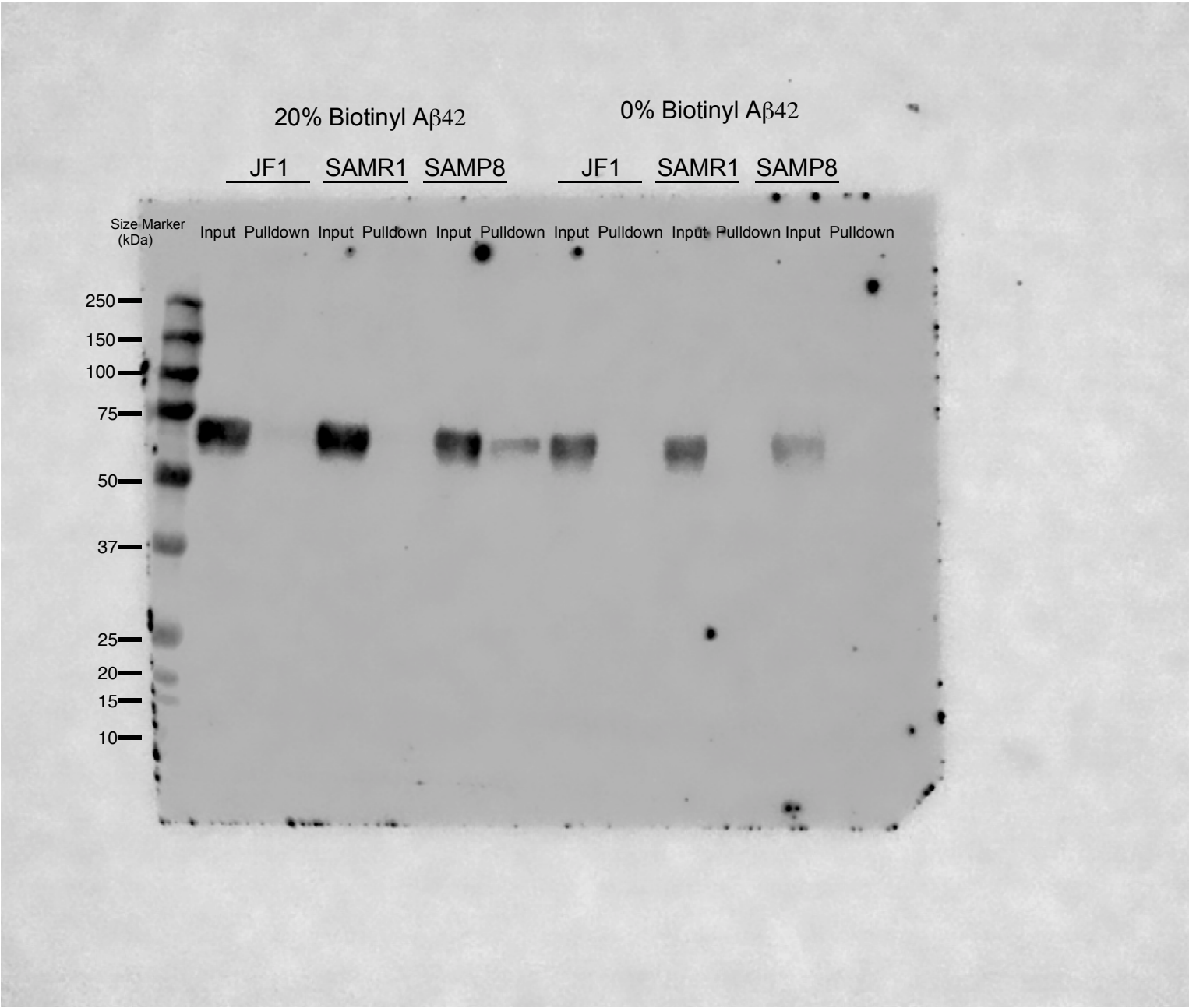

Supplement: S1 Raw images — (PDF) [file pone.0248027.s007.pdf]
