# Supplementary material for: Polymorphic SERPINA3 prolongs oligomeric state of amyloid beta
Source: PLoS One. 2021 Mar 4;16(3):e0248027. doi: 10.1371/journal.pone.0248027 (PMC7932536; doi:10.1371/journal.pone.0248027)
Supplement: S1 Table — (PDF) [file pone.0248027.s006.pdf]

S1 Table. Position of polymorphism of JF1, SAMR1 and SAMP8 type Serpina3n proteins in the open reading frame (ORF).

Serial No.	Amino acid position in ORF	JF1	SAMR1	SAMP8
1.	69	Lysine	Threonine	Threonine
2.	84	Valine	Leucine	Leucine
3.	85	Methionine	Valine	Valine
4.	156	Arginine	Lysine	Lysine
5.	157	Alanine	Threonine	Threonine
6.	258	Phenylalanine	Serine	Serine
7.	273	Methionine	Methionine	**Leucine**
8.	281	Lysine	Lysine	**Arginine**
